# SARS-CoV-2 activates the TLR4/MyD88 pathway in human macrophages: A possible correlation with strong pro-inflammatory responses in severe COVID-19

**DOI:** 10.1016/j.heliyon.2023.e21893

**Published:** 2023-11-17

**Authors:** Sabina Sahanic, Richard Hilbe, Christina Dünser, Piotr Tymoszuk, Judith Löffler-Ragg, Dietmar Rieder, Zlatko Trajanoski, Anne Krogsdam, Egon Demetz, Maria Yurchenko, Christine Fischer, Michael Schirmer, Markus Theurl, Daniela Lener, Jakob Hirsch, Johannes Holfeld, Can Gollmann-Tepeköylü, Carl P. Zinner, Alexandar Tzankov, Shen-Ying Zhang, Jean-Laurent Casanova, Wilfried Posch, Doris Wilflingseder, Guenter Weiss, Ivan Tancevski

**Affiliations:** a Department of Internal Medicine II, Medical University of Innsbruck, Innsbruck, Austria; b Institute of Bioinformatics, Biocenter, Medical University of Innsbruck, Innsbruck, Austria; c Centre of Molecular Inflammation Research, Norwegian University of Science and Technology, Trondheim, Norway; d The Central Norway Regional Health Authority, St. Olavs Hospital HF, Trondheim, Norway; e Department of Internal Medicine III, Medical University of Innsbruck, Innsbruck, Austria; f Department of Cardiac Surgery, Medical University of Innsbruck, Innsbruck, Austria; g Institute of Medical Genetics and Pathology, University Hospital Basel, Basel, Switzerland; h Laboratory of Human Genetics of Infectious Diseases, Necker Branch, INSERM U1163, Necker Hospital for Sick Children, Paris, France; i University of Paris, Imagine Institute, Paris, France; j St. Giles Laboratory of Human Genetics of Infectious Diseases, Rockefeller Branch, The Rockefeller University, New York, NY, USA; k Howard Hughes Medical Institute, New York, NY, 10065, USA; l Division of Hygiene and Medical Microbiology, Medical University of Innsbruck, Austria

**Keywords:** SARS-CoV-2, Macrophages, Innate immunity, Toll-like receptors, COVID-19

## Abstract

**Background:**

Toll-like receptors (TLRs) play a pivotal role in the immunologic response to severe acute respiratory syndrome coronavirus 2 (SARS-CoV-2) infection. Exaggerated inflammatory response of innate immune cells, however, may drive morbidity and death in Coronavirus disease 19 (COVID-19).

**Objective:**

We investigated the engagement of SARS-CoV-2 with TLR4 in order to better understand how to tackle hyperinflammation in COVID-19.

**Methods:**

We combined RNA-sequencing data of human lung tissue and of bronchoalveolar lavage fluid cells derived from COVID-19 patients with functional studies in human macrophages using SARS-CoV-2 spike proteins and viable SARS-CoV-2. Pharmacological inhibitors as well as gene editing with CRISPR/Cas9 were used to delineate the signalling pathways involved.

**Results:**

We found TLR4 to be the most abundantly upregulated TLR in human lung tissue irrespective of the underlying pathology. Accordingly, bronchoalveolar lavage fluid cells from patients with severe COVID-19 showed an NF-κB-pathway dominated immune response, whereas they were mostly defined by type I interferon signalling in moderate COVID-19. Mechanistically, we found the Spike ectodomain, but not receptor binding domain monomer to induce TLR4-dependent inflammation in human macrophages. By using pharmacological inhibitors as well as CRISPR/Cas9 deleted macrophages, we identify SARS-CoV-2 to engage canonical TLR4-MyD88 signalling. Importantly, we demonstrate that TLR4 blockage prevents exaggerated inflammatory responses in human macrophages infected with different SARS-CoV-2 variants, including immune escape variants B.1.1.7.-E484K and B.1.1.529 (omicron).

**Conclusion:**

Our study critically extends the current knowledge on TLR-mediated hyperinflammatory responses to SARS-CoV-2 in human macrophages, paving the way for novel approaches to tackle severe COVID-19.

**Take-home message:**

Our study combining human lung transcriptomics with functional studies in human macrophages clearly supports the design and development of TLR4 - directed therapeutics to mitigate hyperinflammation in severe COVID-19.

## Highlights

• TLR4 is the most abundantly upregulated TLR in human lung irrespective of the underlying pathology.
• BALF MΦ from patients with severe COVID-19 show strong upregulation of the NF-κB pathway.
• The ectodomain of the SARS-COV-2 spike protein leads to TLR4 dependent upregulation of IL-6.
• Viable SARS-CoV-2 leads to TLR4 dependent IL-6 upregulation irrespective of the variant of concern.
• TLR4 inhibition in SARS-CoV-2 infected MΦ leads to downregulation of genes involved in leukocyte migration.

## Abbreviations

ACE2: Angiotensin converting enzyme 2
ALI: Acute lung injury
AMΦ: Alveolar macrophages
BALF: Bronchoalveolar lavage fluid
BSL-3: Biosafety level 3
COVID-19: Coronavirus disease 19
CRISPR: Clustered Regularly Interspaced Short Palindromic Repeats
EBV: Epstein-Barr virus
FCS: Fetal calf serum
HMGB1: High-Mobility Group Box 1
HSV: Herpes Simplex Virus
IFITM3: Interferon Induced Transmembrane Protein 3
IFN: Interferon
IL-1b: Interleukin 1 beta
IL-6: Interleukin 6
IL-6R: Interleukin 6 receptor
ISG: Interferon-stimulated genes
JAK: Janus kinase
MOI: Multiplicity of infection
MyD88: Myeloid differentiation primary response 88
MΦ: Macrophages
NF-κB: Nuclear factor 'kappa-light-chain-enhancer' of activated B-cells
OxPAPC: Oxidized 1-palmitoyl-2-arachidonyl-*sn*-glycero-3-phosphorylcholine
PAMP: Pathogen-associated molecular patterns
PMA: Phorbol 12-myristate-13-acetate
qRT-PCR: Quantitative real-time PCR
RNA: Ribonucleic acid
RNA-Seq: RNA-sequencing
SARS-CoV-2: Severe acute respiratory syndrome coronavirus 2
SARS-CoV-2-ECD: SARS-CoV-2 ectodomain
SARS-CoV-2-RBD: SARS-CoV-2 receptor binding domain
SBP1: Spike binding protein 1
SD: Standard deviation
SgRNA: Single guide RNA
TLR: Toll-like receptor
TMPRSS2: Transmembrane protease serine subtype 2
TRAF 6: TNF receptor associated factor 6
TRIF: TIR-domain-containing adapter-inducing interferon-β

## 1 Introduction

As of August 18, 2023 approximately 769 million people have been infected with the severe acute respiratory syndrome coronavirus 2 (SARS-CoV-2), accounting for seven million deaths. The spike glycoprotein is the main determinant of the virus’ tropism and binds with its subunit S1 to the host entry receptor angiotensin converting enzyme 2 (ACE2). Consecutively the spike protein is cleaved by TMPRSS2, a transmembrane serine protease, which activates the trimers of the second subunit S2 and leads to the fusion of viral and host cell lipid bilayers. This allows the release of viral ribonucleic acid (RNA) into the cell. After the entry, the production of viral proteins is directly initiated, leading to the induction of multiple inflammatory cascades, which depending on the cell type include the production of anti-viral type I interferons (IFNs) or pro-inflammatory cytokines. These cytokines also promote the development of adaptive B- and T-cells. Only a few days after infection B-cells start producing antibodies, which after somatic hypermutation, can last for months and lead to protection against re-infection and/or fatal disease courses. Not only different vaccines have been developed in order to create and boost immunity, but also immunotherapeutic interventions have made a significant contribution to the treatment of acute SARS-CoV-2 infection [[1], [2], [3], [4], [5], [6], [7], [8]].

The acute phase of disease presents within a broad clinical spectrum ranging from mild to fatal disease courses. Clinical data indicate that a hyperinflammatory response (an excessive release of pro-inflammatory cytokines e.g. interleukin 6 (IL-6) and IL-1b with secondary tissue damage) to SARS-CoV-2, contributes to disease severity and death in patients affected by COVID-19 [9]. Conversely, early IL-6R, IL-1b and Janus Kinase (JAK) blockade as well as systemic glucocorticoid therapy were shown to improve the outcome in severely affected patients [[10], [11], [12], [13], [14]].

Toll-like receptors (TLRs) are evolutionary conserved pathogen recognition receptors that play a pivotal role in innate immunity. During acute infection, TLRs sense different pathogen-associated molecular patterns (PAMPs), resulting in the synthesis of various pro-inflammatory cytokines [15]. There is emerging evidence that direct activation of TLR4 in alveolar macrophages (AMΦ) by the SARS-CoV-2 spike protein may play a pivotal role in strong pro-inflammatory responses and associated acute lung injury (ALI) observed in severe COVID-19 [16,17]. *In silico* molecular docking experiments suggested that SARS-CoV-2 Spike protein may directly bind TLR4, which was recently confirmed by two independent laboratories, both in murine and human monocytes and MΦ [[16], [17], [18]]. In the healthy human lung, TLR4 is mainly expressed in AMΦ (www.proteinatlas.org). Besides direct activation by SARS-CoV-2 Spike protein, endogenous TLR4 ligands, which might be released upon severe lung tissue damage, may include surfactant-derived oxidized phospholipids, S100A8/A9, High-Mobility Group Box 1 (HMGB1), proteoglycans and Surfactant protein A [[19], [20], [21], [22], [23]]. These properties make TLR4 a pivotal switch for the immediate engagement of inflammatory circuits in lungs exposed to SARS-CoV-2 and prone to ALI. A workflow scheme of our study has been incorporated in the Supplementary information, Fig. S1.

Here, we report that the SARS-CoV-2 markedly enhances pro-inflammatory cytokine production in human macrophages via TLR4, irrespective of the variant of concern.

## 2 Materials and methods

### 2.1 Cell lines and culture

The human monocytic THP-1 cells (American Type Culture Collection, Manassas, Va) were differentiated into macrophages by culturing with 50 ng/mL of phorbol 12-myristate 13-acetate (PMA; Merck, 16561-29-8) in RPMI supplemented with 10 % heat-inactivated fetal calf serum (FCS) and antibiotics for 2 days [24].

For the deletion of TLR4 (TLR4^*del*^) and MyD88 (MyD88^*del*^) in THP-1 cells, we employed the lentiviral CRISPR/Cas9 system developed by the Zhang laboratory [25]. The respective plasmids were provided by Mariya Yurchenko. Lentiviral production, transduction and selection of the target cells were done according to the methods described in her paper [26]. The guide sequences for the sgRNAs encoded in the plasmids are as follows; TLR4: gCCAGCTTTCTGGTCTCACGC, MyD88: gCTCCGCCAGCGCGGTCCAGT. Lower case letters denote 5′ inserted guanosines that are not part of the target sequence, but a prerequisite for proper transcription by the U6 promoter. The deletion was confirmed using Western blot as well as FACS analysis (Supplementary information, Figs. S2 and S3). Western blot analysis was performed according to standard protocols (Bio-Rad Laboratories) and as previously published by our group [27]. Briefly, isolated cells were lysed in RIPA buffer (50 mM Tris, pH 7.4, 150 mM NaCl, 1 % Nonidet P-40, 0.5 % sodium deoxycholate, and 0.1 % SDS) and supplemented with protease inhibitors (Thermo Fisher Scientific, 78443). Protein quantity was determined by Bradford assay (Bio-Rad Laboratories, 5000006) and equal amounts of protein were denatured at 70 °C in Laemmli buffer with reducing agent, resolved on SDS-PAGE and transferred to a polyvinylidene fluoride membrane (Sigma, GE10600023). After blocking the membrane in 5 % skimmed milk, primary antibody (MyD88 Rabbit PolyAB, proteintech) was incubated over night at 4 °C. Signal was visualized with HRP-conjugated secondary antibodies (Agilent) and ECL Select Western Blotting Detection Reagent (Amersham, RPN2235). Densitometry of immunoblots was performed with ImageJ. Viability analysis were performed using the Alamar Blue® Assay (AB, Bio-Rad, BUF012A) as follows: resazurin yields a fluorescent product after reduction in living cells. The AB assay was carried out according to manufacturer's instructions. Control medium was removed, the cells were rinsed with PBS and 200 μl of AB solution (10 % of AB prepared in fresh FCS free medium) were added to each well. 500 μg/mL Zeocin served as a positive control. After 3 h of incubation the fluorescence was measured at the respective excitation and emission wavelength of 540 and 595 nm using a Tecan Spark® microplate reader. No significant differences in viability between wildtype and TLR4^*del*^ THP-1 cells were observed (Supplementary information, Fig. S4A). For proliferation analysis, BrdU labelling was performed. Both, wildtype and TLR4^*del*^ THP-1 cells were pulsed with 10 μM BrdU (Sigma-Aldrich) 16 h before harvesting. Intracellular staining for BrdU was performed using the BD Pharmingen™ FITC BrdU Flow Kit (BD, 559619) according to manufacturer's instructions. Consecutively, cells were analysed by the use of flow cytometry. Both, wildtype and TLR4^*del*^ THP-1 cells showed >90 % proliferating cells (Supplementary information, Fig. S4B).

Human embryonic kidney (HEK293FT, Invitrogen) cells, stably expressing huACE2 under a doxycycline-inducible promoter (pPB-TA-ACE2-ERP2) were established using the PiggyBac transposon system (pCS-hypPBase). For the construction of the PiggyBac transposase plasmid, we used the sequence published by Yusa et al., flanked by attB sites, and synthetized by idtDNA as a gBlock [28]. This gBlock was introduced into pDONR207 (Invitrogen) employing the Gateway BP (Invitrogen) reaction, and subsequently introduced into the final constitutive expression vector pCS-Dest (Addgene, #22423) by Gateway cloning (LR reaction). This new plasmid was termed pCS-hypPBase and employed for stable introduction of PiggyBac repeat - containing plasmids into cell lines. Human ACE2 (huACE2) was amplified by PCR from pCEP4-myc-ACE2 by adding flanking attB sites (Addgene #141185). This PCR product was purified and first introduced into pDONR207, then into the pPB-TA-ERP2 (Addgene #80477) by Gateway cloning, resulting in the doxycycline inducible construct pPB-TA-ACE2-ERP2.

HEK293FT cells were seeded on the day before transfection at a density of 5 × 10^5^ cells/well in DMEM (4,5 g/L glucose) culture medium supplemented with 10 % FCS and antibiotics. Cells were subsequently transfected with equal amounts of pCS-hypPBase and pPB-TA-huACE2-ERP2 using Transporter 5 transfection reagent (Polysciences, 26008) according to the manufacturer's protocol. After a two-week selection process using 5 μg/ml Puromycin (ThermoScientific, A1113803), huACE2 expression was induced with 1.25 μM doxycycline for 24 h. As shown in Supplementary information, Fig. S5AB, SARS-CoV-2-ECD led to a reduction of ACE2 in HEK293FT cells constitutively overexpressing human ACE2, reflecting canonical ACE2 downregulation and shedding.

Human A549 alveolar epithelial (A549, ATCC CCL-185) and Chinese hamster ovary-K1 (CHO–K1, ATCC CCL-61) cells were cultured according to ATCC's protocol.

### 2.2 Reagents

In order to selectively block TLR4 signalling in cells, the inhibitor TAK-242 (Merck, 614316) was used at 10 μM [29]. The angiotensin converting enzyme 2 (ACE2) activity inhibitor MLN-4760 (Merck, 530616) was employed at 10 μM [30], the TLR3 inhibitor (R)-2-(3-Chloro-6-fluorobenzo[b]thiophene-2-carboxamido)-3-phenylpropanoic acid (Merck, 614310) at 30 μM [31], the TIR-domain-containing adapter-inducing interferon-β (TRIF) inhibitor peptide (pepinh-TRIF, InvivoGen, tlrl-pitrif) at 5 μM (manufacturer's instruction), the Myeloid differentiation primary response 88 (MyD88) inhibitor peptide NBP2-29328 (Novus Biologicals, NBP2-29328) at 10 μM, the TNF receptor associated factor 6 (TRAF6) inhibitor C25-140 (MedChem Express, HY-120934) at 30 μM [32], the TMPRSS2 inhibitor Camostat mesylate (TOCRIS, 3193) at 10 μM [33], the Cathepsin B and L inhibitor E−64d (TOCRIS, 4545) at 10 μM [34], the spike binding protein (SBP1, TOCRIS, 7233) at 10 μM (manufacturer's instruction), the TLR7/9 inhibitor hydroxychloroquine sulfate (MedChem Express, HY-B1370) at 12 μM [35]. Lipopolysaccharide (LPS, 10 ng/ml, Sigma, L2630) and Poly I:C (20 μg/ml, TOCRIS, 4287) served as a positive control for TLR4-and TLR3-dependent inflammation. The differentially induced IL-6 expression in THP-1 MΦ by LPS and Poly I:C is depicted in Supplementary information, Fig. S6. The final concentrations of vehicles (H_2_O, dimethyl sulfoxide, ethanol) were equivalent (less than 0.1 % of the culture medium) in all experiments. By performing dose-response studies, TLR4 and TLR3 pathway inhibitors were confirmed to adequately inhibit LPS (TLR4) or Poly I:C (TLR3) – induced inflammation, respectively (Supplementary information, Fig. S7). TLR4 inhibitor off-target effects were precluded as follows. Addition of different TLR2 agonists (TLR2/1 agonist: Pam3CSK4 10 ng/mL and TLR2/6 agonist: Pam2CSK4 10 ng/mL, InvivoGen, tlrl-pms and tlrl-pm2s-1) to THP-1 MΦ resulted in TLR2-mediated IL-6 upregulation, yet the co-incubation with the employed TLR4 inhibitor TAK-242 failed to suppress IL-6 expression (Supplementary information, Fig. S8). In addition, all inhibitors were tested for cytotoxicity using the aforementioned Alamar Blue® Assay, 5 % ethanol served as positive control (Supplementary information, Fig. S9).

### 2.3 Synthesis of recombinant SARS-CoV-2 spike receptor binding domain (RBD) and –ectodomain (ECD)

Cloning and production of tagged spike protein constructs were performed according to previously published protocols [36,37]. In short, gBlocks with codon-optimised attB flanked sequences coding for the mentioned proteins were ordered from idtDNA and cloned into expression vectors (pPB-TA-ERP2). Transfected CHO–K1 cells excreted the proteins of interest into the cell culture supernatant, which was subsequently purified with the ÄKTA chromatography system. The aforementioned vectors expressed either the trimeric ectodomain (aa13-1213, ECD) or the receptor binding domain (aa333-529, RBD) of the SARS-CoV-2 spike protein under a doxycycline inducible promoter for protein production. The RBD protein sequence was fused with the FC domain of mouse IgG2a and combined with a strong artificial secretion signal peptide at the N-terminus [38]. Instead of the FC domain we used a twin strep tag for Spike-ECD for easier purification. The subsequent cloning procedure was identical to pPB-TA-huACE2-ERP2, resulting in the expression plasmids pPB-TA-SARS-CoV-2-ECD-ERP2 (ectodomain) and pPB-TA-SARS-CoV-2-RBD–FC–ERP2 (receptor binding domain). For the production of the SARS-CoV-2-ECD and SARS-CoV-2-RBD, we established two stable CHO–K1 (ATCC CCL-61) cell lines with the PiggyBac system. For this purpose, we used the pPB-TA-SARS-CoV-2-ECD-ERP2 or the pPB-TA-SARS-CoV2-RBD–FC–ERP2 plasmid and employed a similar transfection and selection strategy as in the HEK293FT_TA-huACE2-ERP2 cells (please refer to *Cell lines and culture*). Puromycin (15 μg/ml) - selected cells were grown in 175 cm flasks. The supernatant was harvested twice a week and immediately frozen at −80 °C. For protein purification of SARS-CoV-2-RBD-FC, we established a 2-step chromatography protocol using a HiTrap Phenyl FF column (Cytiva) followed by a Protein-A affinity chromatography column (J.T. Baker Bakerbond PROchivA) on an Äkta go (Cytiva) system. For SARS-CoV-2-ECD a one-step protocol with the Strep-Tactin®XT 4Flow® high capacity FPLC column (iba) was sufficient. After elution from the respective columns SARS-CoV-2 spike proteins were concentrated using Amicon Ultra-15 and washed 3–4 times with 15 ml protein buffer and adjusted to a concentration of 5 mg/ml. Consecutive purity analysis was performed on SDS-page as described above. Potential endotoxin contamination was ruled out by the Pierce™ Chromogenic Endotoxin Quant Kit (A39552, Thermo Fisher) according to manufacturer's manual. SARS-CoV-2-RBD and -ECD were shown to be endotoxin-free, with endotoxin levels of 4.6*10^−3^ endotoxin units (EU)/μg and 16.5*^10^−3^ EU/μg respectively, which is far below the commonly used cut-off of <0.1 μg/mg protein endotoxin (<1 EU/μg).

### 2.4 Virus expansion and infection of THP-1 cells

SARS-CoV-2 ancestral strain (Wuhan-Hu-1), Alpha (B.1.1.7) and Beta (B.1.351) were retrieved from repositories (BEI Resources, Manassas, VA, USA; CFAR/NIBSC; Nr-52281, Nr-52282, NR-52286) and SARS-CoV-2 Alpha-E484K (B1.1.7.-E484K), Delta (B.1.617.2) and Omicron (B.1.1.529/BA1) were isolated from clinical specimens of SARS-CoV-2 positive swabs (Ethics statement, ECS1166/2020) [39].

Viruses were propagated as previously published by our laboratories [40] and according to the manufacturer's instructions in Vero/TMPRSS2-expressing cells and used subsequently to infect human THP-1 macrophages, ensuring endotoxin-free levels below the detection limit of 0.005 U/ml (0.0005 ng/ml). VeroE6/TMPRSS2 is an engineered VeroE6 cell line expressing high levels of TMPRSS2, making these cells highly susceptible to SARS-CoV-2 infection and propagation. The cell line was obtained via the CFAR (NIBSC) and is described in Matsuyama et al. [41] After expansion, SARS-CoV-2 was absolutely quantified using a SARS-CoV-2 specific qRT-PCR; infectivity was determined by plaque assays [42,43]. Human THP-1 macrophages were infected at indicated multiplicity of infection (MOI). All experiments with viable virus were performed in Biosafety level 3 (BSL-3) laboratories.

#### 2.4.1 RNA isolation, reverse transcription, and TaqMan quantitative real-time PCR (qRT-PCR)

Total RNA was extracted using PeqGold Trifast (VWR) according to the manufacturer's protocol and reverse transcribed with M-MLV Reverse Transcriptase (Thermo Fisher Scientific). For qRT-PCR experiments we used the QuantStudio5 real-time PCR system (Thermo Fisher Scientific) and either SsoFast EvaGreen Supermix (for reactions without fluorescent probes) or SsoAdvanced Universal Probes Supermix (for those with probes) [44].

The following primer sequences were used (sequences from 5′ to 3’).

### 2.5 IL1beta

Fwd CTG CTC TGG GAT TCT CTT CAG.

Rev ATC TGT TTA GGG CCA TCA GC.

### 2.6 IL6

Fwd AGC CCA CCG GGA ACG AAA GAG A.

Rev AAG GCA GCA GGC AAC ACC AGG.

Probe FAM-AAC TCC TTC TCC ACA AGC GCC TTC-BHQ1.

### 2.7 Tubulin

Fwd TCCTTCAACACCTTCTTCAGTGAG AC G.

Rev GGTGCCAGTGCGAACTTCATCA.

Probe FAM-ATGTGCCCCGGGCAGTGTTTGTAGACTTG-BHQ1.

### 2.8 ISG15

Fwd ACTCATCTTTGCCAGTACAGG.

Rev CAGCTCTGACACCGACATG.

### 2.9 IFITM1

Fwd ATCAACATCCACAGCGAGAC.

Rev CAACCATCTTCCTGTCCCTAG.

### 2.10 IFITM2

Fwd GATGTCCACCGTGATCCAC.

Rev CAACCATCTTCCTGTCCCTAG.

### 2.11 IFITM3

Fwd GTCCACCGTGATCCACATC.

Rev CAACCATCTTCCTGTCCCTAG.

### 2.12 MX1

Fwd GAAGATAAGTGGAGAGGCAAGG.

Rev CTCCAGGGTGATTAGCTCATG.

### 2.13 Human IL-6 ELISA

Human IL-6 protein levels were measured using a commercially available ELISA kit with a detection sensitivity of 4 pg/mL (Human IL-6 ELISA Set, Lot. Nr: 44086, BD Biosciences). The immunoassay was performed according to the manufacturer's instructions.

### 2.14 Fluorescence-activated cell sorting

Human ACE2 transfected HEK293FT cells were incubated with SARS-CoV-2-ECD as previously described [45]. Cells were harvested, washed and labelled with huACE2 antibody (1:100, AC384 Novus Biologicals); Alexa Flour 647 anti-mouse IgG1 was used as secondary antibody (1:100, BioLegend, 406617). Flow cytometry measurements were performed on a Beckman-Coulter Gallios device and analysed with the FlowJo software (FlowJo LLC). Cell viability was assessed using DAPI (Thermo Fisher Scientific) [44].

### 2.15 Histological staining and immunohistochemistry

For histological analysis, lung explant specimen from a patient undergoing lung transplantation due to severe COVID-19 associated lung damage were used. Paraffin-embedded lung sections (1–2 μm) were deparaffinised using a xylol and ethanol series followed by target retrieval procedure with citrate buffer (Dako, S1699) by autoclavation for 15 min at 121 °C. Samples were blocked in blocking/staining solution (5 % goat serum, 0.2 % Triton X-100, 0.2 % bovine serum albumin in phosphate buffered saline) for 1 h at room temperature. Primary antibody incubation was performed overnight at 4 °C using an anti-CD68-antibody (Abcam, ab125047) diluted 1:100. Goat anti-rabbit Alexa Fluor 488 - labelled IgG served as secondary antibody (1:500, Thermo Fisher, A11008). A monoclonal mouse anti-TLR4 antibody (1:100, Abcam ab22048) was incubated at 37 °C for 30 min, followed by 3 washing steps and incubation with the secondary antibody goat anti-mouse Alexa Fluor 594 (1:500, Thermo Fisher, A11032) for 30 min at 37 °C. Finally, sections were washed and embedded using a fluorescence mounting medium (Dako, S3023) containing DAPI (0.1 μg/ml, Thermo Fisher, 62248) and analysed on an Olympus VS120 device [44].

### 2.16 TLR expression in autoptic lungs

All GEP work was performed by HTG according to established protocols (https://www.htgmolecular.com/assets/htg/resources/BR-05-HTG-EdgeSeq-System.pdf) [46,47]. In brief, lysates from samples were run on the HTG EdgeSeq Processor (HTG Molecular Diagnostics, Tucson, AZ, USA) using the HTG EdgeSeq Immune Response Panel with an excess of nuclease protection probes (NPPs) complimentary to their target. S1 nuclease then removed un-hybridized probes, and RNAs leaving behind NPPs hybridized to their targets in a 1-to-1 ratio. Samples were individually barcoded using a 16-cycle PCR reaction to add adapters and molecular barcodes, individually purified using AMPure XP beads (Beckman Coulter, Brea, CA, USA) and quantified using a KAPA Library Quantification kit (KAPA Biosystems, Wilmington, MA, USA). Libraries were sequenced on the Illumina SEQUENCER platform (Illumina, San Diego, CA, USA) for quantification. Quality control, standardization and normalization were performed by HTG and provided to the investigators. Quality control criteria by the manufacturer were that the percentage of overall reads allocated to the positive process control probe per sample is less than 28 %, the read depth is at least 750,000 and the relative standard deviation of reads allocated to each probe within a sample is greater than 0.094. In addition, only samples with a known post-mortem interval were preserved for differential analysis. Differential expression analysis was conducted in R version 4.0.3 (R Project for Statistical Computing, Vienna, Austria) with the DESeq2 package using default settings. Count estimates were normalized with the median ratio method. Prior to supervised heatmap visualization, i.e. according to the predefined disease and control groups, the normalized counts were further $log_2$ transformed using a robust variance stabilization. The heatmap of TLR genes was produced with the complexHeatmap package. Column sample clusters were obtained by k-means clustering with k = 2 and row gene clusters by hierarchical clustering with complete linkage.

### 2.17 Single-cell RNA-sequencing data analysis in BALF cells from COVID-19 patients

Single-cell sequencing data provided by Liao et al. were analysed with R programing suite version 4.0.3 and tidyverse package bundle as previously described [48,49]. KEGG pathway enrichment analysis with significantly regulated transcripts was performed with kegga tool from limma R package for genes significantly differentially regulated in bronchoalveolar lavage fluid macrophages [50].

### 2.18 Bulk RNA-sequencing data analysis in THP-1 cells

RNA was isolated as described above. RNA integrity was verified with the Agilent Bioanalyzer. Ion TorrentTM compatible libraries were generated from 500 ng total RNA input, using the QuantSeq 3′ mRNA-Seq Library Prep Kit from Lexogen (Lexogen, Vienna Biocenter, Austria). Barcoded libraries were multiplexed and sequenced on the Ion TorrentTM Proton Sequencer (Ion torrent, Thermo Fisher Scientific). The Ion Torrent Suite was used for splitting of reads into barcodes, and initial quality filtering and adapter trimming, resulting in a final output of an average 7 million reads per sample. Subsequently, all sequencing reads were trimmed to a maximum length of 320 bases using fastp [51]. We then ran the nf-core/rnaseq (version 3.1) pipeline to align the resulting reads to the human genome (GRCh38) with STAR and to assess the read counts on the gene models from GENCODE version 33 with Salmon [[52], [53], [54]]. Differential expressed genes were calculated using DESeq2 (version 1.32.0) using a fold change threshold of 1.5 and a FDR of 0.1 [55]. GO and Pathway overrepresentation- or GSEA analyses were performed with the R bioconductor package ClusterProfiler (version 4.0) [56]. We used the R package EnhancedVolcano to generate volcano plots and labelled down-regulated genes of the NF-kappa B signalling pathway (KEGG hsa04064).

### 2.19 Ethics

All studies on human specimen were performed in accordance with the Declaration of Helsinki and the European Data Policy. All participants gave written informed consent. Isolation of viral strains from clinical specimens was granted approval from the ethics committee at the Medical University Innsbruck (1166/2020). Autoptic patient sample and data collection was approved by the Ethics committee of Northern and Central Switzerland (study ID 2020-00969), and the COVID-19 collective has been previously reported [57]. For the histological analysis from a patient undergoing lung transplantation due to severe COVID-19 associated lung damage informed consent for analysis and publication was obtained.

### 2.20 Data analysis and statistics

Data were analysed using GraphPad Prism version 8.0 (GraphPad Software, San Diego, CA). All experiments were repeated at least three times. Continuous variables with normal distribution are expressed as the mean ± standard deviation (SD). Significance was determined by Mann-Whitney *U* test (non-parametric distribution) or unpaired two-tailed student's t-test (for pairwise comparisons, parametric distribution) and one-way ANOVA (for comparisons among three or more groups, parametric distribution). The post hoc test was performed with Tukey's multiple comparisons following ANOVA. Asterisks indicate statistical significance (*P < 0.05; **P < 0.01; ***P < 0.001). Data are shown as means ± SD (n ≥ 3).

## 3 Results

### 3.1 The TLR4 - NF-κB pathway is central to SARS-CoV-2 hyperinflammation

**Fig. 1:**
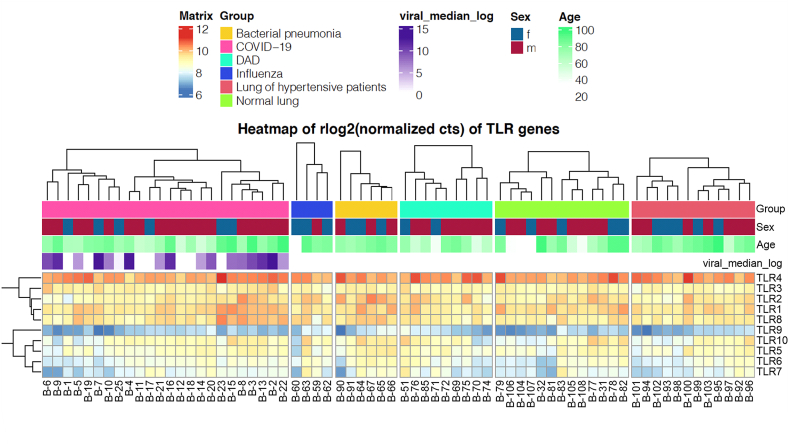
Heatmap of TLR RNA expression in human lung autopsy samples determined by HTG EdgeSeq Technology. (DAD: diffuse alveolar damage; f: female; m: male).

In the healthy human lung, TLR4 is mainly expressed in AMΦ (http://www.proteinatlas.org/) Analogously, we demonstrate co-staining of MΦ and TLR4 in lung explant specimens from a patient with end-stage COVID-19 related lung injury (Supplementary information, Fig. S10). To extend this knowledge, we used autoptic lung samples from patients (n = 68) died of infectious diseases, such as bacterial pneumonia (n = 6/68), influenza (n = 4/68) and COVID-19 (n = 24/68), as well as autopsies from patients with diffuse alveolar damage (n = 9/68) and hypertension (n = 12/68) (Supplementary information, Table S1). Autoptic samples from healthy lungs served as a control (n = 13/68). Using RNA-sequencing (RNA-Seq, HTG EdgeSeq Technology), we identified TLR4 as the most abundantly expressed TLR in the human lung, irrespective of the underlying pulmonary condition (Fig. 1), whereas TLR9 showed the weakest expression in human lung tissue.

Employing a single-cell RNA-Seq approach in bronchoalveolar lavage fluid (BALF) from patients with severe COVID-19, Liao et al. previously found that both AMΦ and newly recruited monocyte-derived MΦ are major drivers of local inflammation in the lung, similarly to what has been described in African green monkeys and in K18-hACE2-transgenic mice [48,58,59]. Data in mice indicate that succumbing AMΦ are gradually replaced by Ly6C^+^ monocytes, creating a highly inflammatory microenvironment in the lung [59]. To investigate the engagement of TLR4 pathways in SARS-CoV-2 mediated activation of MΦ in the human lung, we next interrogated the single-cell RNA-Seq dataset from BALF in patients affected by COVID-19 published by Liao et al. [48] In patients with moderate COVID-19, KEGG pathway enrichment analysis showed a strong upregulation of Herpes simplex virus (HSV) 1 infection pathways, which is heavily relying on type I IFN response (Supplementary information, Fig. S11A), whereas ribosomal activity (reflecting overall protein translation) and COVID-19 related pathways are shut down, the latter encompassing ACE2, TMPRSS2, NRP1, ADAM17, IL-6R and the JAK-STAT as well as TLR2/4 dependent IL-6 amplifier [60]. In contrast, in BALF MΦ from patients with severe COVID-19, our signalling pathway enrichment analysis showed strong upregulation of the NF-κB pathway, together with two NF-κB response amplifiers: the Epstein Barr Virus (EBV) pathway engaging TLR2-MyD88, and the NOD2 pathway which serves as RNA sensor for *Coronaviridae* (Supplementary information, Fig. S11B) [61,62].

### 3.2 The ectodomain of SARS-CoV-2 spike protein induces inflammation in human MΦ via TLR4

To analyse the response of TLR4 to SARS-CoV-2 spike protein, we established the synthesis of SARS-CoV-2-RBD and SARS-CoV-2-ECD. The purity of both spike protein subunits was confirmed by electrophoresis analysis (Supplementary information, Fig. S12).

**Fig. 2:**
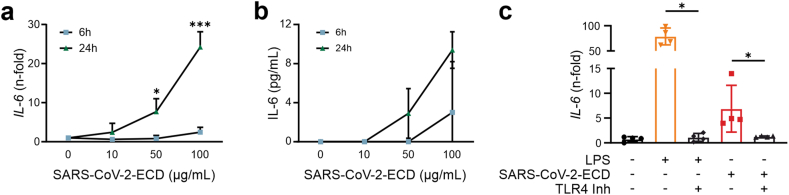
THP-1 MΦ were incubated with increasing concentrations of SARS-CoV-2-ECD for 6h and 24h. IL-6 RNA (**A**) and (**B**) protein levels in supernatants were quantified by qRT-PCR and ELISA, respectively; **C:** qRT-PCR analysis of IL-6 in THP-1 MΦ incubated with TLR4 ligand LPS (10 ng/mL), with SARS-CoV-2-ECD (100 μg/mL), with and without the TLR4 inhibitor TAK-242 (10 μM) for 24h. *P < 0.05, ***P < 0.001; n = 4. Graphs show mean ± SD (TLR4: toll-like receptor 4; SARS-CoV-2-ECD: Severe acute respiratory syndrome coronavirus 2 ectodomain).

THP-1 MΦ were challenged with increasing concentrations of both spike protein subunits for six and 24 h, after which mRNA levels of *IL-6* were measured by qRT-PCR. SARS-CoV-2-ECD significantly induced *IL-6* in a time- and dose-dependent manner, whereas the treatment with SARS-CoV-2-RBD did not alter *IL-6* transcription. (Fig. 2A and Supplementary information, Fig. S13). The pro-inflammatory effect of SARS-CoV-2-ECD was verified on the protein level by the use of ELISA, showing markedly increased IL-6 levels in MΦ supernatants (Fig. 2B). Importantly, induction of *IL-6* expression in human MΦ by SARS-CoV-2-ECD was blocked by concomitant treatment with a selective TLR4 inhibitor, LPS served as control (Fig. 2C)

### 3.3 TLR4 as a target to prevent hyperinflammation in MΦ infected with viable SARS-CoV-2

**Fig. 3:**
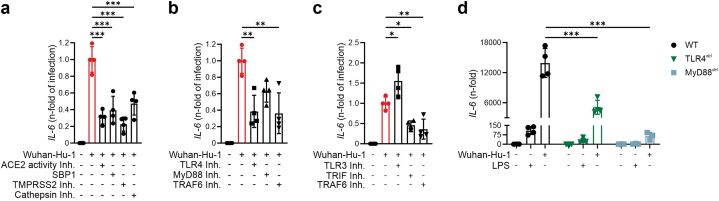
Human THP-1 MΦ infected with viable SARS-CoV-2 (Wuhan-Hu-1, MOI 0.005) and treated with inhibitors of SARS-CoV-2 entry (**A**), of the TLR4 (**B**) and of the TLR3 pathway (**C)**. **D**: Wildtype, TLR4 deleted and MyD88 deleted THP-1 MΦ were infected with viable SARS-CoV-2 (Wuhan-Hu-1, MOI: 0.005) and TLR4 agonist LPS (10 ng/mL). After 24h, IL-6 expression was determined by qRT-PCR. For detailed description of inhibitors used, see methods section. *P < 0.05; **P < 0.01; ***P < 0.001; n = 4. Graphs show mean ± SD. (del.: deleted; ACE2: angiotensin converting enzyme 2; SBP1: spike binding protein 1; TMPRSS2: transmembrane protease serine subtype 2; TLR: toll-like receptor; MyD88: Myeloid differentiation primary response 88; TRAF6: TNF receptor associated factor 6; TRIF: TIR-domain-containing adapter-inducing interferon-β).

Whereas our data confirm that the Spike protein induces SARS-CoV-2-associated hyperinflammation in MΦ by binding to TLR4, infection with viable SARS-CoV-2 may cause a more exaggerated TLR4-mediated response or possibly engage with additional inflammatory pathways, such as TLR3 and TLR7/9 mediated responses through cellular pathogen recognition of viral and/or cellular RNA. Thus, we next studied the impact of viable SARS-CoV-2 (strain Wuhan-Hu-1) on the inflammatory response in human THP-1 MΦ. As shown in Supplementary information, Fig. S14A, viable SARS-CoV-2 dramatically induced the expression of *IL-6* in a dose-dependent manner. Inhibition of viral entry (SBP1, TMPRSS2 inhibitor and cathepsin inhibitor) as well as of ACE2 activity reduced SARS-CoV-2-induced *IL-6* expression by more than 50 % (Fig. 3A). Importantly, the use of a selective TLR4 inhibitor as well as of a TRAF6 inhibitor reduced SARS-CoV-2-induced *IL-6* expression by >60 % and >40 %, respectively (Fig. 3B). TRIF and TRAF6 are shared between TLR3 and TLR4 signalling. Whereas both adaptors were found to be involved in SARS-CoV-2-associated hyperinflammation, inhibition of TLR3 did not reduce *IL-6* expression (Fig. 3C). Analogously, inhibition of TLR7/9 did not reduce SARS-CoV-2 mediated expression of *IL-6* (Supplementary information, Fig. S13B). Importantly, similar effects were observed for *IL-1b* expression, another gene induced via the classical TLR4-MyD88 cascade (Supplementary information, Fig. S15AB). On the contrary, the infection of human MΦ with SARS-CoV-2 did not lead to the production of type I *IFNs* or *ISGs*, indicating a subordinated anti-viral role of MΦ during acute infection (Supplementary information, Fig. S16).

**Fig. 4:**
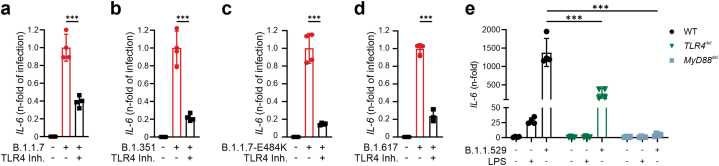
THP-1 MΦ infected with viable SARS-CoV-2 variants of concern, MOI: 0.005: **(A)** B.1.1.7., **(B)** B.1.351, **(C)** B.1.1.7.-E484K (**D**) B.1.617 and treated with TLR4 inhibitor TAK-242 (10 μM). **E**: Wildtype, TLR4 deleted and MyD88 deleted THP-1 MΦ were infected with viable SARS-CoV-2 omicron variant (B.1.1.529, MOI: 0.005) and TLR4 agonist LPS (10 ng/mL). After 24h, IL-6 expression was quantified by qRT-PCR. Graphs show mean ± SD. (del.: deleted; TLR: toll-like receptor; MyD88: Myeloid differentiation primary response 88).

To confirm the central role of the TLR4-MyD88 pathway in SARS-CoV-2 induced hyperinflammation, we infected THP-1 MΦ with SARS-CoV-2 in which either TLR4 or MyD88 had been deleted by the use of CRISPR/Cas9 technology. Both TLR4^*del*^ and MyD88^*del*^ MΦ were protected from an exaggerated immune response elicited by SARS-CoV-2 (Fig. 3D). Finally, our approach targeting TLR4 dramatically suppressed the hyperinflammatory response also in human MΦ infected with different variants of concern according to the CDC (B.1.1.7, B.1351, B.1.1.7-E484K, B.1.617 and B.1.1.529) (Fig. 4A–E).

### 3.4 RNA-Seq analysis of SARS-CoV-2 infected MΦ reveals translational potential of TLR4 inhibition for patients affected by severe COVID-19

**Fig. 5:**
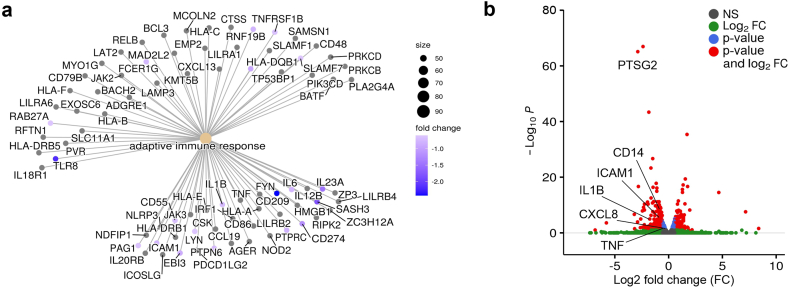
**A:** GO enrichment analysis of THP-1 MΦ infected with viable SARS-CoV-2 (Wuhan-Hu-1, MOI 0.005) with and without TLR4 inhibitor TAK-242 (10 μM). Blue-coloured dots indicate genes downregulated by TAK-242. **B:** Volcano plot of differentially expressed genes in THP-1 MΦ infected with viable SARS-CoV-2 (Wuhan-Hu-1, MOI 0.005) and treated with the TLR4 inhibitor TAK-242 (10 μM), highlighting downregulated genes of the NF-κB signalling pathway (KEGG database). (fold-change cut-off: 1.5, adjusted p-value cut-off: 0.01) (NS: not significant; FC: fold-change)

Finally, to better anticipate the therapeutic potential of TLR4 inhibition, we performed RNA-Seq analysis in human THP-1 MΦ infected with SARS-CoV-2. As shown in Fig. 5A and Supplementary information, Fig. S17, TLR4 inhibition blunted the expression of several immune response genes, as well as of genes regulating leukocyte cell-cell-interaction, locomotion, migration and motility in THP-1 MΦ. Eventually, RNA-Seq analysis of genes involved in the TLR4-NF-κB pathway not only corroborated inhibition of SARS-CoV-2 induced hyperinflammation (*IL1B*, *TNF*, *PTGS2*, *CXCL8*, *ICAM1),* but also unveiled downregulation of *CD14* expression, the co-receptor of TLR4 (Fig. 5B). Taken together, these findings suggest pleiotropic effects of TLR4 inhibition in human MΦ, indicating novel potential therapeutic options for the treatment of patients affected by severe COVID-19.

## 4 Discussion

Innate immunity represents the first line of defence against SARS-CoV-2 infection. The interplay between SARS-CoV-2 infected cells and immune cells has been described as a tightly orchestrated, spatio-temporal process leading to macrophage extravasation as disease progresses [63,64]. In particular, type I and III interferons play a pivotal role in inhibiting the viral entry into the cytosol and virus replication. Recent studies by our COVID human genetic effort consortium (COVIDhge, http://www.covidhge.com) and by others showed that the anti-viral response may be blunted by several mechanisms such as autoantibodies neutralizing type I IFNs, inborn errors of type I IFN immunity and changes in the Fc component of SARS-CoV-2-directed antibodies [[65], [66], [67], [68], [69], [70]]. Further evidence of imbalanced host response to SARS-CoV-2 comes from studies profiling transcriptomes and proteomes of COVID-19 patients [71,72]. These data revealed that besides low levels of type I and III IFNs, elevated chemokines and cytokines, such as IL-6, IL-1β, IL-1RA, IL-8 (CXCL8), IL-18, and TNF-α account for disease severity and death in COVID-19, indicating a sepsis-like response upon progression of disease [73].

In line, by mining the RNA-Seq library published by Liao et al., we found that the transcriptome of lung MΦ is dominated by an adequate IFN response in moderate COVID-19 cases, whereas it shifts to pro-inflammatory NF-kB - signatures in severe cases. These findings are in line with our COVIDhge data [67,69,70,74], again showing that an adequate type I IFN response is crucial to counteract SARS-CoV-2 infection and COVID-19 progression, and highlighting the association of an exaggerated immune response with a severe course of disease. Besides high activity of the NF-κB pathway, our signalling analysis showed engagement of the EBV pathway in BALF MΦ from patients with severe COVID-19, which is characterized by CD21^−^, CD19^−^and MHC-II dependent entry mechanisms, and by a TLR2-MyD88 dependent additional activation of NF-κB [75]. Interestingly, linear regression analyses showed IFITM3 to be upregulated in human BALF MΦ from patients affected either by moderate or severe COVID-19 (data not shown), which may well reflect anti-viral type I IFN response. However, this did not hold true in human THP-1 MΦ, where we measured the expression of several ISGs including IFITM3 upon SARS-CoV-2 infection, indicating that IFN response in human lung MΦ observed *in vivo* may rather be the consequence of transcellular IFN signalling.

A central constituent of the innate immunity driving inflammatory responses in sepsis is the Toll-like receptor 4 [76], and we and others present different lines of evidence to ascribe a crucial role to TLR4 in the development of ALI in COVID-19 [16,17]; **I)** TLR4 constitutes a major sensor for pathogens as well as for endogenous alarmins including oxidized surfactant-derived phospholipids, and its activation in AMΦ was previously shown to promote hyperinflammation and ALI in mice [19]. Moreover, we show **II)** that TLR4 is the most abundantly expressed TLR in the human lung, and **III)** that its expression is merely restricted to AMΦ in severe COVID-19.

Whereas others already showed that the Spike protein may serve as TLR4 ligand [16,17], we demonstrate for the first time that viable SARS-CoV-2 induces hyperinflammation in human MΦ via TLR4 and extend the available knowledge by the notion that human TLR4, in contrast to ACE2, is engaged only by the full extracellular domain of SARS-CoV-2 and not by the RBD [77]. We also show that TLR4-mediated inflammation in human MΦ is not restricted to the ancestral SARS-CoV-2 variant, but extends to any of the tested variants of concern, clearly constituting a so far under-recognized major virulence factor of the novel *Coronavirus*.

Treatment with a specific TLR4 inhibitor markedly reduced the expression of *IL-6* and of *IL-1b* via the MyD88 pathway, indicating significant therapeutic potential of targeting TLR4 in severe SARS-CoV-2. Complementing our results, a previous study has found that TLR4 deficient mice were resistant to acid- and H5N1-induced acute lung injury [19]. In another study, infection of *Tlr4*^*−/−*^ mice with the phylogenetically related SARS-CoV resulted in significantly higher virus replication and disease severity compared to wildtype mice [78]. The anti-inflammatory role of TLR4 has been discussed in several studies, and a common concept infers that TLR4 ligands may potentiate type I IFN production in plasmacytoid dendritic cells, which are the major source for IFN [69,79]. The current data thus suggests that systemic blockade of TLR4 may come to the cost of attenuated IFN-mediated immunity during early virus infection. Available data also suggest that, similarly to what observed for IL-6R/IL-1R blockade [80,81] and systemic corticosteroid treatment [82], a TLR4-blocking therapeutic may critically prevent hyperinflammation in the lung and thereby ALI if administered at a later time point when exaggerated immune-mediated inflammation prevails. Alternatively, TLR4 in AMΦ may be directly targeted via an inhaled route of administration [83].

In line with our results, Zheng et al. recently showed that *TLR4* and *MyD8*8 RNA expression correlated with COVID-19 disease severity in patients [84]. In addition, they showed that one major driver of SARS-CoV-2 associated hyperinflammation may be the binding of its E protein to MΦ TLR2. Complementing these results, our analysis of RNA-Seq data in BALF from severe COVID-19 patients showed an upregulation in the Epstein-Barr virus infection pathway, which is known to involve TLR2-MyD88 signalling. In their study, the authors used the lipid 1-palmitoyl-2-arachidonyl-*sn*-glycero-3-phosphorylcholine (OxPAPC) to block TLR2. OxPAPC, in turn was previously shown to form from surfactant during ALI, and was discussed to further potentiate inflammation and thereby ALI by activating TLR4 on AMΦ [19,84]. However, OxPAPC was also found to effectively inhibit both, TLR2 and TLR4, in human THP-1 MΦ via the binding of the co-receptor CD14 and MD2 [85]. Yet, the knowledge about immunological effects of OxPAPC remains controversial, as other studies have indicated that it may function as a TLR4 and TLR2 agonist both *in vitro* and *in vivo*, overall limiting its potential as drug to inhibit hyperinflammation in severe COVID-19 [19,[85], [86], [87], [88]].

In addition, different TLR based therapeutics have already been developed and are currently undergoing clinical trials [[89], [90], [91]]. Importantly, a monoclonal antibody blocking TLR4 was recently shown to reduce mortality at 28 days in severe COVID-19 by 66.0 % when compared to placebo on top of standard of care [92]. Our findings may critically contribute to unveil the exact mechanisms underlying the observed beneficial effect of this antibody.

Overall, we found that inhibition and/or genetic loss of MyD88 in human MΦ, a crucial adaptor protein for both TLR2 and TLR4, showed the most significant reduction in pro-inflammatory cytokines upon SARS-CoV-2 infection. These data indicate a redundancy between TLR2 and TLR4 in the development of SARS-CoV-2 induced hyperinflammation in human MΦ, where both pathogen recognition receptors might synergistically act as viral sensors, depending on whether the E or the Spike protein are available as ligands.

Our study bears limitations. Due to the limited resources available to our research team, we were not able to conduct animal experiments for this study. Specifically, we lacked access to BSL-3 animal facilities, which are required for working with SARS-CoV-2. As a result, we were limited to *in vitro* experiments and computational analyses, which may not fully reflect the complexities of biological systems *in vivo*. While this represents a limitation of our study, we believe that by our broad approach, combining human transcriptomic data with functional data *in vitro*, our findings still provide valuable insights into the mechanisms underlying our research question.

In summary, the response of TLR4 and of TLR2 to SARS-CoV-2 may switch the anti-viral response of the lung from the release of type I IFNs to an exaggerated synthesis of pro-inflammatory mediators, explaining at least in part the hyperinflammation observed in severe COVID-19.

The concept of TLR2 and TLR4 activation through SARS-CoV-2 has been recently described, complementing the paradigmatic view that SARS-CoV-2 leads to exaggerated inflammation mainly through ACE2 downregulation and shedding. We extend this knowledge by showing for the first time that viable SARS-CoV-2 engages the TLR4-MyD88 pathway in human MΦ, and that TLR4 inhibition dramatically suppresses the hyperinflammatory response irrespective of the SARS-CoV-2 variant. We conclude that the evolutionary advantage of TLR4 improved defence against infections might come to the cost of increased risk for SARS-CoV-2 associated hyperinflammation and death, constituting a model of pleiotropic antagonism.

## Data availability

The data that support this study's findings are available from the corresponding authors (I.T., D.W. W.P.) upon request.

## CRediT authorship contribution statement

**Sabina Sahanic:** Conceptualization, Data curation, Formal analysis, Investigation, Methodology, Project administration, Resources, Validation, Visualization, Writing – original draft, Writing – review & editing. **Richard Hilbe:** Data curation, Formal analysis, Methodology, Writing – review & editing. **Christina Dünser:** Data curation, Formal analysis, Writing – review & editing. **Piotr Tymoszuk:** Data curation, Formal analysis. **Judith Löffler-Ragg:** Conceptualization, Resources, Supervision, Validation, Writing – review & editing. **Dietmar Rieder:** Data curation, Formal analysis, Investigation, Methodology, Validation, Visualization, Writing – review & editing. **Zlatko Trajanoski:** Data curation, Formal analysis, Investigation, Validation, Writing – review & editing. **Anne Krogsdam:** Data curation, Formal analysis, Methodology, Writing – review & editing. **Egon Demetz:** Data curation, Formal analysis, Writing – review & editing. **Maria Yurchenko:** Methodology, Resources, Writing – review & editing. **Christine Fischer:** Data curation, Writing – review & editing. **Michael Schirmer:** Resources, Validation, Writing – review & editing. **Markus Theurl:** Methodology, Resources, Writing – review & editing. **Daniela Lener:** Data curation, Methodology, Writing – review & editing. **Jakob Hirsch:** Methodology, Writing – review & editing. **Johannes Holfeld:** Methodology, Resources, Writing – review & editing. **Can Gollmann-Tepeköylü:** Methodology, Resources, Writing – review & editing. **Carl P. Zinner:** Data curation, Formal analysis, Investigation, Methodology, Writing – review & editing. **Alexandar Tzankov:** Data curation, Formal analysis, Methodology, Resources, Writing – review & editing. **Shen-Ying Zhang:** Investigation, Validation, Writing – review & editing. **Jean-Laurent Casanova:** Methodology, Validation, Writing – review & editing. **Wilfried Posch:** Data curation, Methodology, Resources, Validation, Writing – review & editing. **Doris Wilflingseder:** Formal analysis, Methodology, Resources, Validation, Writing – review & editing. **Guenter Weiss:** Resources, Supervision, Writing – review & editing. **Ivan Tancevski:** Conceptualization, Formal analysis, Methodology, Project administration, Supervision, Validation, Writing – original draft, Writing – review & editing.

## Declaration of competing interest

The authors declare the following financial interests/personal relationships which may be considered as potential competing interests.
