# Supplementary material for: SARS-CoV-2 activates the TLR4/MyD88 pathway in human macrophages: A possible correlation with strong pro-inflammatory responses in severe COVID-19
Source: Heliyon. 2023 Nov 17;9(11):e21893. doi: 10.1016/j.heliyon.2023.e21893 (PMC10686889; doi:10.1016/j.heliyon.2023.e21893)
Supplement: Multimedia component 1 [file mmc1.docx]

Supplementary information

**SARS-CoV-2 activates the TLR4/MyD88 pathway in human macrophages: a possible correlation with strong pro-inflammatory responses in severe COVID-19**

Sabina Sahanic, MD^1^, Richard Hilbe, BSc^1^, Christina Dünser, MSc^1^, Piotr Tymoszuk, PhD^1^, Judith Löffler-Ragg, MD^1^, Dietmar Rieder, PhD^2^, Zlatko Trajanoski, PhD^2^, Anne Krogsdam, PhD^2^, Egon Demetz, PhD^1^, Maria Yurchenko, PhD^3,4^, Christine Fischer, MSc^1^, Michael Schirmer, MD^1^, Markus Theurl, MD^5^, Daniela Lener, BSc^5^, Jakob Hirsch, MD^6^, Johannes Holfeld, MD^6^, Can Gollmann-Tepeköylü, MD^6^, Carl Zinner, PhD^7^, Alexandar Tzankov, MD^7^, Shen-Ying Zhang, PhD^8,9,10^, Jean-Laurent Casanova, MD^8,9,10,11^, Wilfried Posch, PhD^12^*, Doris Wilflingseder, PhD^12^*, Guenter Weiss, MD^1^, Ivan Tancevski, MD^1^*

^1^Department of Internal Medicine II, Medical University of Innsbruck, Innsbruck, Austria

^2^Institute of Bioinformatics, Biocenter, Medical University of Innsbruck, Innsbruck, Austria.

^3^Centre of Molecular Inflammation Research, Norwegian University of Science and Technology, Trondheim, Norway

^4^The Central Norway Regional Health Authority, St. Olavs Hospital HF, Trondheim, Norway

^5^Department of Internal Medicine III, Medical University of Innsbruck, Innsbruck, Austria

^6^Department of Cardiac Surgery, Medical University of Innsbruck, Innsbruck, Austria

^7^Institute of Medical Genetics and Pathology, University Hospital Basel, Basel, Switzerland

^8^Laboratory of Human Genetics of Infectious Diseases, Necker Branch, INSERM U1163, Necker Hospital for Sick Children, Paris, France.

^9^University of Paris, Imagine Institute, Paris, France.

^10^St. Giles Laboratory of Human Genetics of Infectious Diseases, Rockefeller Branch, The Rockefeller University, New York, NY, USA.

^11^Howard Hughes Medical Institute, New York, NY, 10065, USA.

^12^Division of Hygiene and Medical Microbiology, Medical University of Innsbruck, Austria

*Correspondence should be addressed to:

1. Asst.-Prof. Dr. Ivan Tancevski

Department of Internal Medicine II, Medical University of Innsbruck

Anichstraße 35, 6020 Innsbruck, Austria

Tel: +43 50504 81602; e-mail: [ivan.tancevski@i-med.ac.at](mailto:ivan.tancevski@i-med.ac.at)

1. Asst.-Prof. Dr. Wilfried Posch

Institute of Hygiene and Medical Microbiology, Medical University of Innsbruck

Schöpfstraße 41/R311, 6020 Innsbruck, Austria

Tel: +43 512 9003-70706; e-mail: [wilfried.posch@i-med.ac.at](mailto:wilfried.posch@i-med.ac.at)

1. Univ.-Prof. Dr. Doris Wilflingseder

Institute of Hygiene and Medical Microbiology, Medical University of Innsbruck

Schöpfstraße 41/R311, 6020 Innsbruck, Austria

Tel: +43 512 9003-70706; e-mail: doris.wilflingseder@i-med.ac.at

**Supplementary Table:**

|  | **Code** | **Sex** | **Age (years)** | **Disease** |  |
| --- | --- | --- | --- | --- | --- |
|  | B-6 | male | 74 | Covid-19 |  |
|  | B-9 | male | 88 | Covid-19 |  |
|  | B-1 | female | 67 | Covid-19 |  |
|  | B-5 | male | 66 | Covid-19 |  |
|  | B-19 | male | 79 | Covid-19 |  |
|  | B-7 | female | 81 | Covid-19 |  |
|  | B-10 | male | 85 | Covid-19 |  |
|  | B-25 | female | 71 | Covid-19 |  |
|  | B-4 | male | 77 | Covid-19 |  |
|  | B-11 | male | 58 | Covid-19 |  |
|  | B-17 | female | 61 | Covid-19 |  |
|  | B-21 | male | 71 | Covid-19 |  |
|  | B-16 | male | 89 | Covid-19 |  |
|  | B-12 | male | 54 | Covid-19 |  |
|  | B-18 | male | 72 | Covid-19 |  |
|  | B-14 | male | 53 | Covid-19 |  |
|  | B-20 | male | 65 | Covid-19 |  |
|  | B-23 | female | 89 | Covid-19 |  |
|  | B-15 | female | 94 | Covid-19 |  |
|  | B-8 | male | 71 | Covid-19 |  |
|  | B-3 | male | 95 | Covid-19 |  |
|  | B-13 | male | 75 | Covid-19 |  |
|  | B-2 | male | 85 | Covid-19 |  |
|  | B-22 | male | 96 | Covid-19 |  |
|  | B-60 | female | 34 | Influenza |  |
|  | B-63 | female | 90 | Influenza |  |
|  | B-59 | male | 171 | Influenza |  |
|  | B-62 | female | 82 | Influenza |  |
|  | B-90 | male | 75 | Bacterial pneumonia |  |
|  | B-91 | female | 71 | Bacterial pneumonia |  |
|  | B-64 | female | 76 | Bacterial pneumonia |  |
|  | B-67 | male | 82 | Bacterial pneumonia |  |
|  | B-65 | female | 86 | Bacterial pneumonia |  |
|  | B-66 | male | 80 | Bacterial pneumonia |  |
|  | B-51 | female | 74 | Diffuse alveolar damage |  |
|  | B-76 | male | 55 | Diffuse alveolar damage |  |
|  | B-85 | male | 67 | Diffuse alveolar damage |  |
|  | B-71 | female | 39 | Diffuse alveolar damage |  |
|  | B-72 | male | 81 | Diffuse alveolar damage |  |
|  | B-69 | male | 84 | Diffuse alveolar damage |  |
|  | B-75 | male | 73 | Diffuse alveolar damage |  |
|  | B-70 | male | 48 | Diffuse alveolar damage |  |
|  | B-74 | male | 62 | Diffuse alveolar damage |  |
|  | B-79 | female | 82 | Normal lung |  |
|  | B-106 | male | 36 | Normal lung |  |
|  | B-104 | male | 34 | Normal lung |  |
|  | B-107 | female | 31 | Normal lung |  |
|  | B-32 | male | 96 | Normal lung |  |
|  | B-81 | male | 73 | Normal lung |  |
|  | B-83 | female | 84 | Normal lung |  |
|  | B-105 | male | 54 | Normal lung |  |
|  | B-108 | male | 39 | Normal lung |  |
|  | B-77 | male | 65 | Normal lung |  |
|  | B-31 | male | 76 | Normal lung |  |
|  | B-78 | female | 92 | Normal lung |  |
|  | B-82 | female | 92 | Normal lung |  |
|  | B-101 | male | 65 | Lung of hypertensive patients |  |
|  | B-94 | male | 70 | Lung of hypertensive patients |  |
|  | B-102 | female | 88 | Lung of hypertensive patients |  |
|  | B-93 | female | 81 | Lung of hypertensive patients |  |
|  | B-98 | female | 71 | Lung of hypertensive patients |  |
|  | B-100 | male | 82 | Lung of hypertensive patients |  |
|  | B-99 | female | 86 | Lung of hypertensive patients |  |
|  | B-103 | male | 91 | Lung of hypertensive patients |  |
|  | B-95 | female | 97 | Lung of hypertensive patients |  |
|  | B-97 | male | 58 | Lung of hypertensive patients |  |
|  | B-92 | male | 79 | Lung of hypertensive patients |  |
|  | B-96 | male | 85 | Lung of hypertensive patients |  |

**Supplementary information, Table S1: Demographics of pulmonary autopsies depicted in Figure 1**

**Supplementary Figures:**


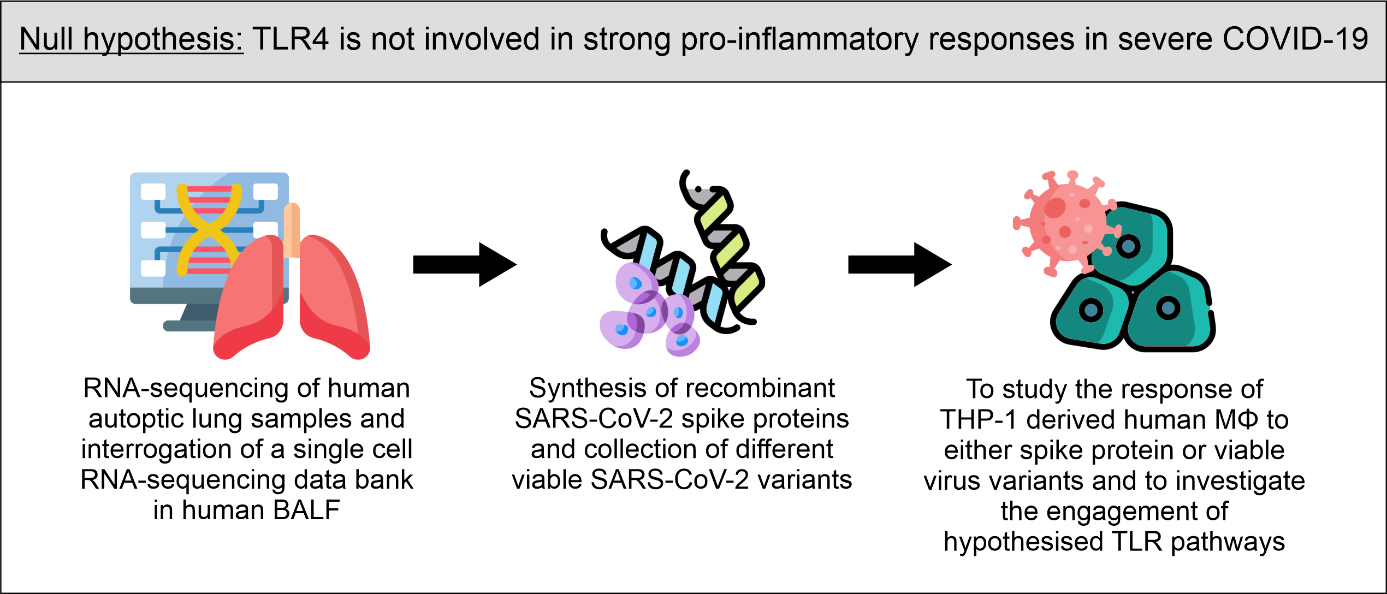


**Supplementary information, Figure S1:** Workflow scheme. Icons were taken from [www.flaticon.com](http://www.flaticon.com)


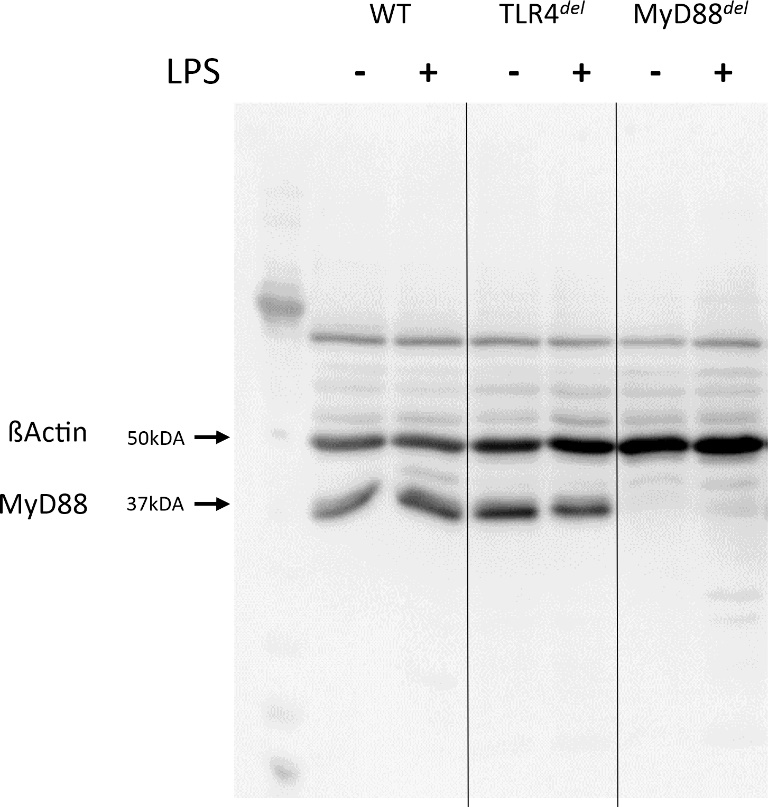


**Supplementary information, Figure S2:** Immunoblot of wildtype (WT) THP-1, *TLR4* deleted THP-1 and *MyD88* deleted THP-1 cells using anti-MyD88 antibody. Beta-Actin served as loading control. Del = deleted


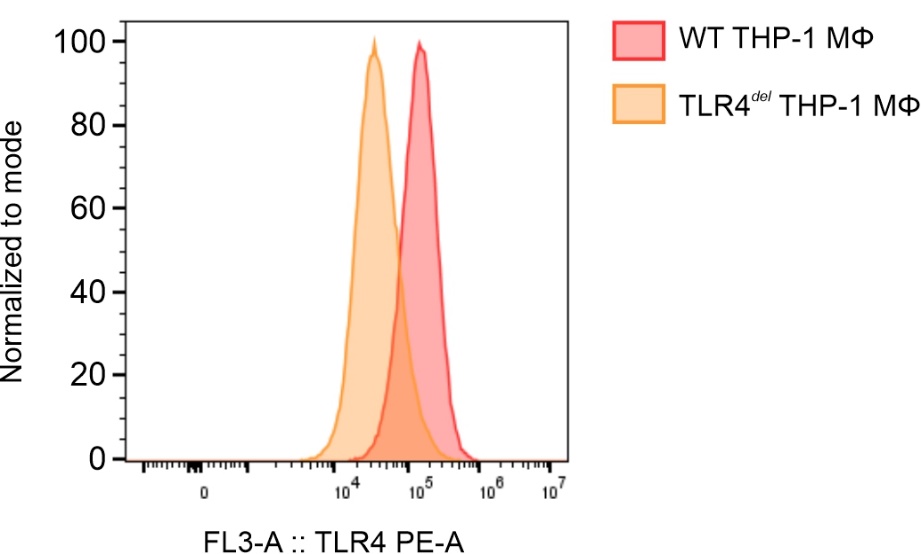


**Supplementary information, Figure S3:** FACS analysis using anti-TLR4-PE-A antibody for quantification of TLR4 surface expression in wildtype and *TLR4* deleted THP-1 derived macrophages. WT = wildtype, TLR4*^del^* = Toll-like receptor 4 deleted


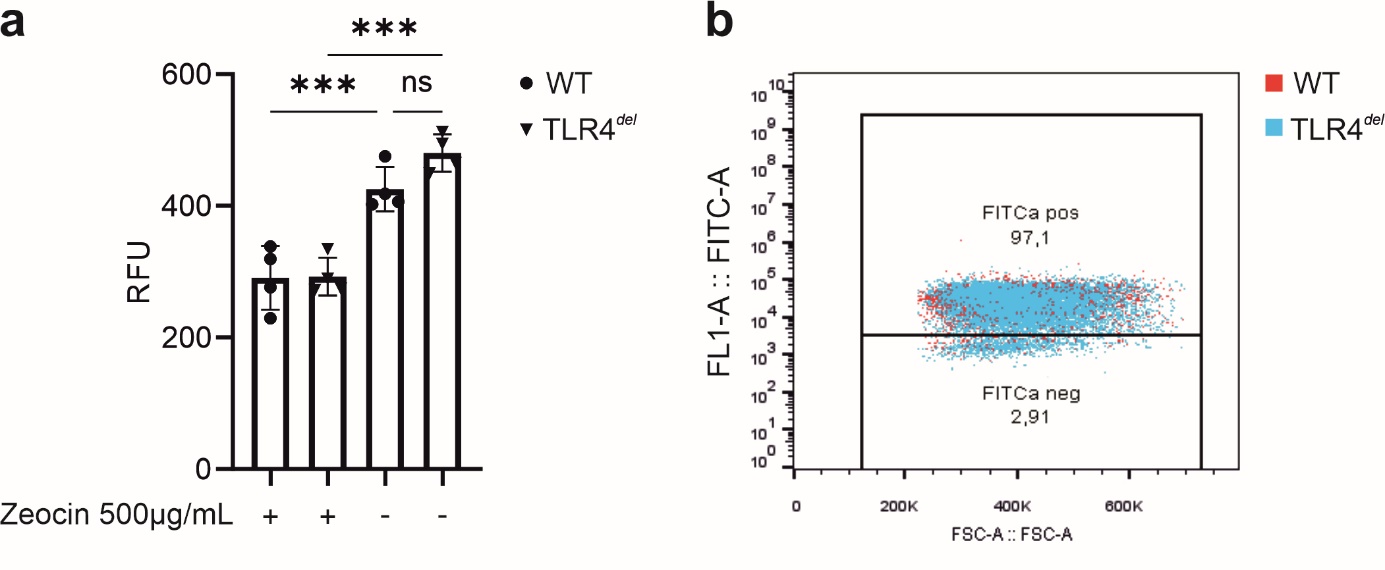


**Supplementary information, Figure S4: (a)** Alamar blue® assay in human wildtype and TLR4 deleted THP-1 cells. Zeocin served as a positive control. **(b)** BrdU proliferation of human THP-1 wildtype and TLR4 deleted cells quantified using FACS analysis after 16 hours of pulsing. ***P<0.001. WT = wildtype, TLR4*^del^* = Toll-like receptor 4 deleted


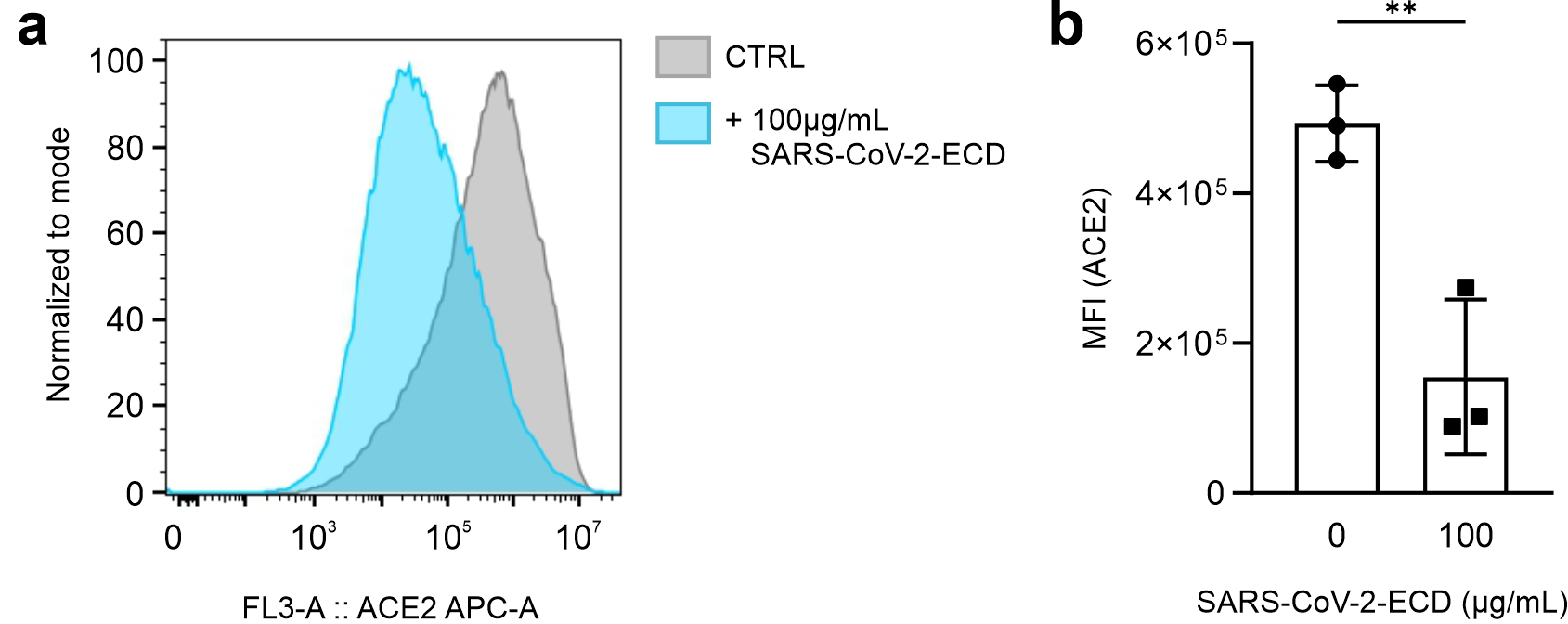


**Supplementary information, Figure S5**: Flow cytometry analysis showing the expression of ACE2 in human ACE2-overexperssing HEK293t cells established in our laboratory, with and without the addition of SARS-CoV-2-ECD. **(a)** Representative histogram, **(b)** Mean fluorescence intensity (MFI) of HEK293t cells without and with SARS-CoV-2-ECD (100µg/mL); **P < 0.01, n=3.

**
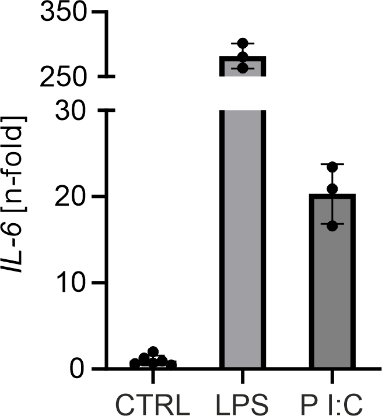
**

**Supplementary information, Figure S6:** Human THP-1 MΦ were incubated with 10ng/mL LPS and 20µg/mL Poly I:C for 24 hours. Expression of *IL-6* was determined by qRT-PCR.


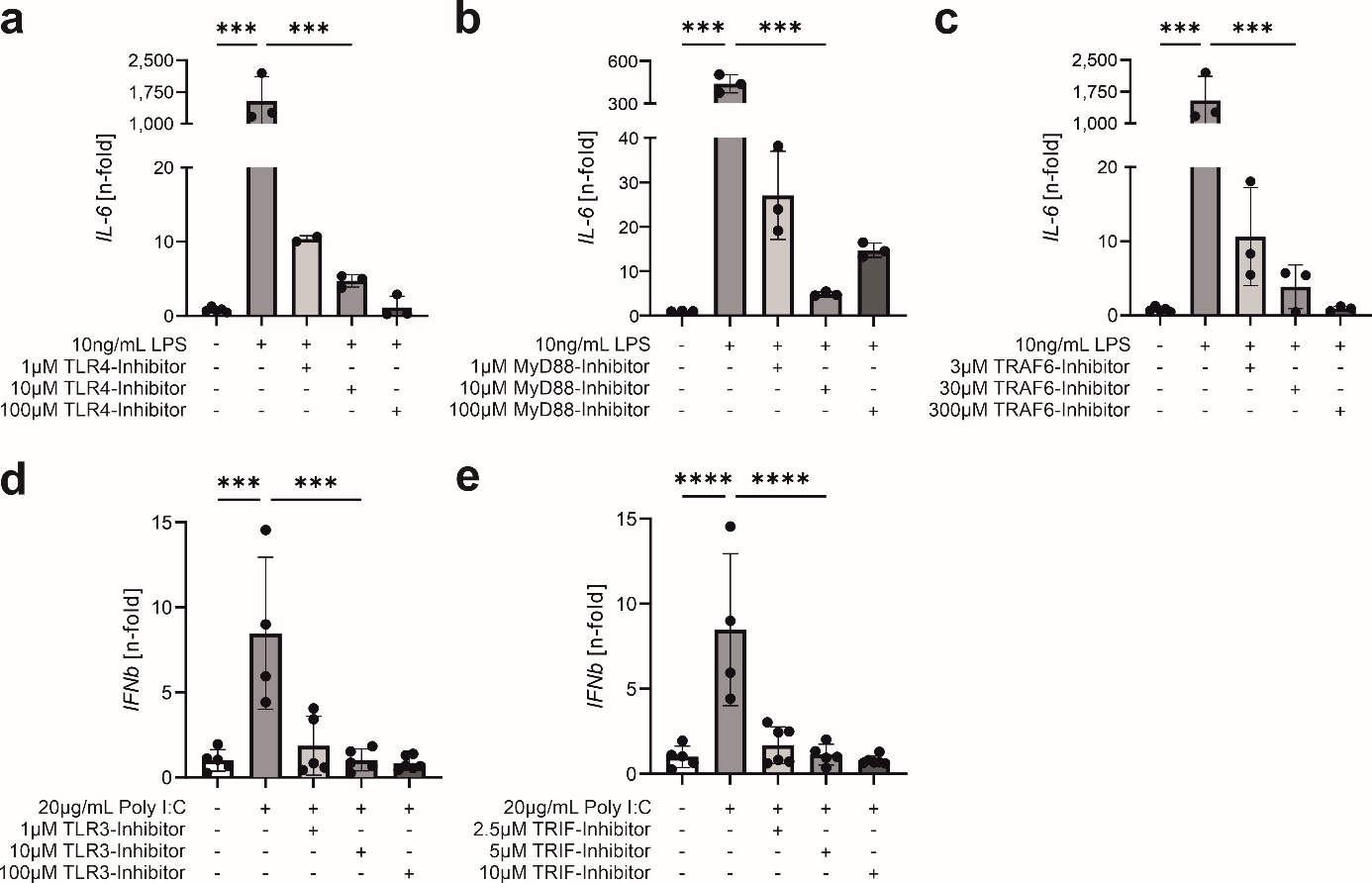


**Supplementary information, Figure S7:** Human THP-1 MΦ (**a-c**) and human A549 epithelial cells (**d-e**) were stimulated with TLR4 agonist LPS (10ng/mL) or TLR3 agonist Poly I:C (20µg/mL) and treated with inhibitors at increasing concentrations. After 24 hours, transcription of target genes *IL-6* and *IFN-b* was quantified by qRT-PCR; n=3.


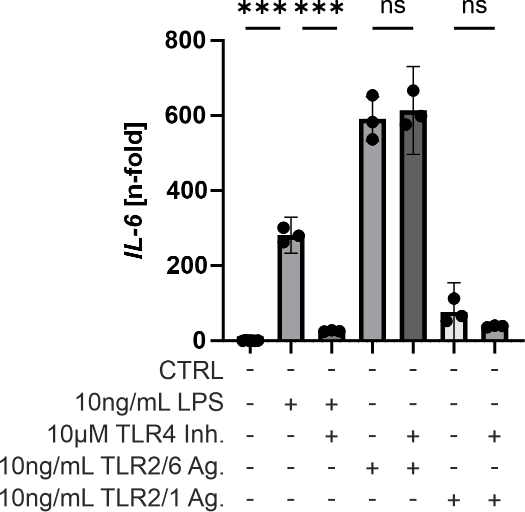


**Supplementary information, Figure S8:** Human THP-1 MΦ were incubated with LPS and TLR2 agonists in the presence and absence of TAK-242. After 24 hours, IL-6 expression was quantified by the use of qRT-PCR; n=3.

**
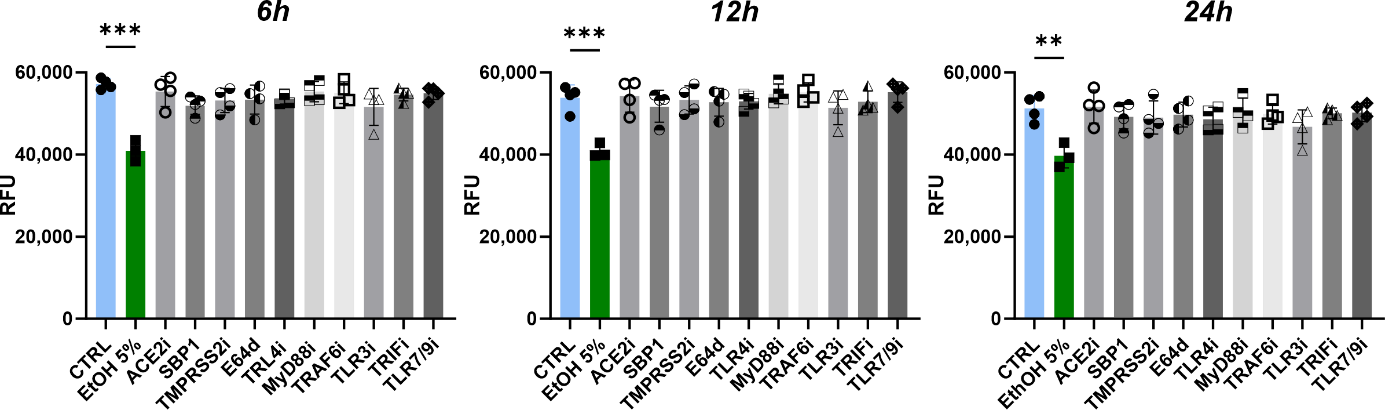
**

**Supplementary information, Figure S9:** Alamar blue® assay for human THP-1 MΦ incubated with indicated chemical TLR pathway inhibitors over a period of 24 hours. Five percent ethanol (EtOH 5%) served as a positive control; n=4. RFU = relative fluorescence units.


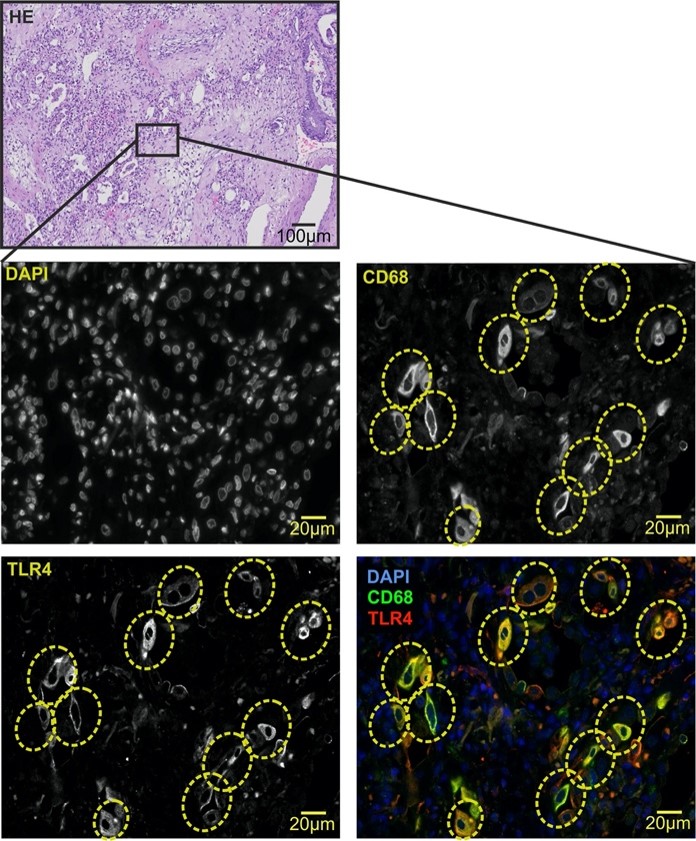


**Supplementary information, Figure S10**: Histological section of human lung explant in a patient undergoing lung transplantation due to severe COVID-19 associated lung damage. TLR4 expression is highest in MΦ visualized by the use of an anti-CD68 Antibody.


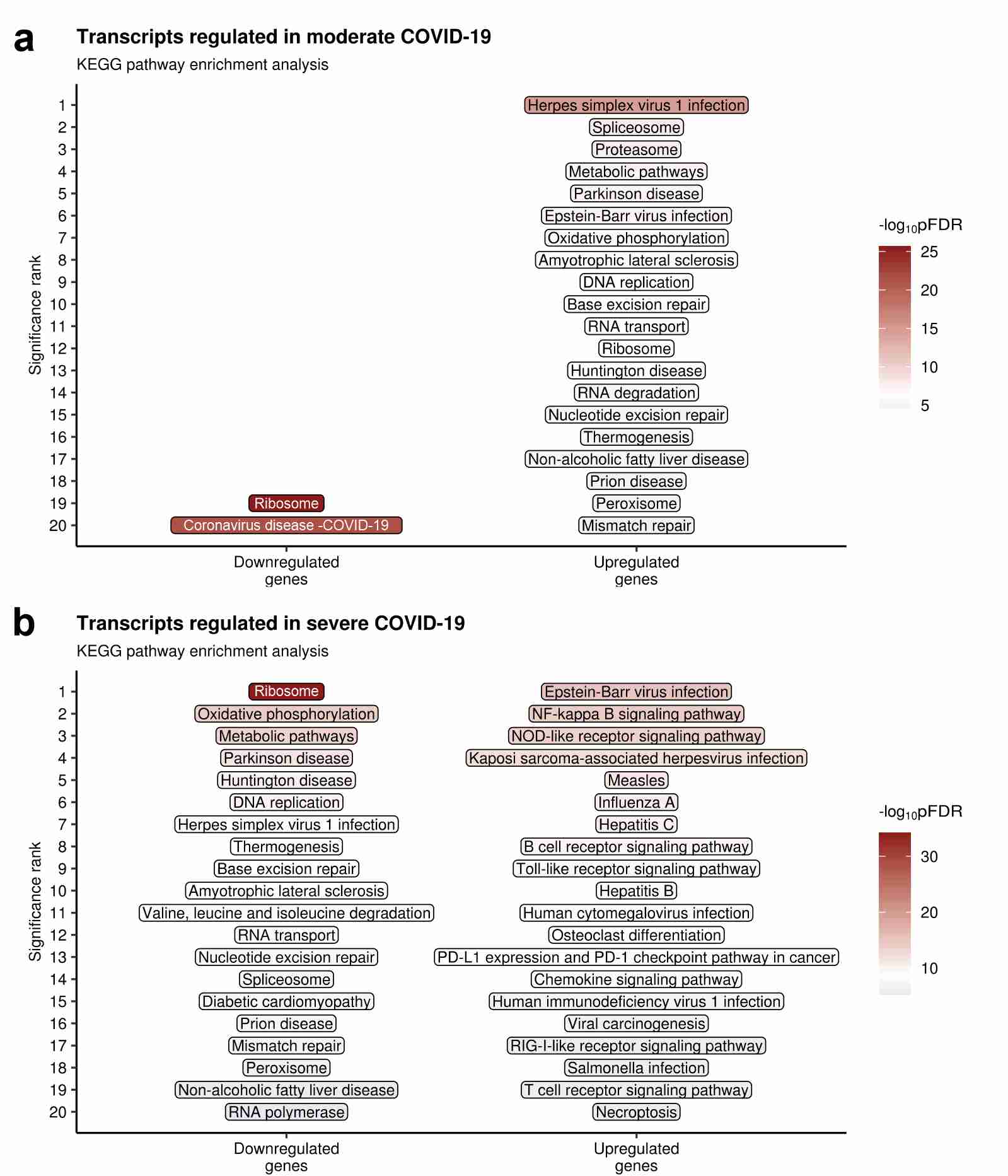


**Supplementary information, Figure S11**: KEGG pathway enrichment analysis done with kegga tool from limma R package for genes significantly differentially regulated in BALF MΦ from patients affected by moderate (**a**) and severe (**b**) COVID-19. Top 20 most significantly enriched pathways in the significantly up- and downregulated gene sets are presented.


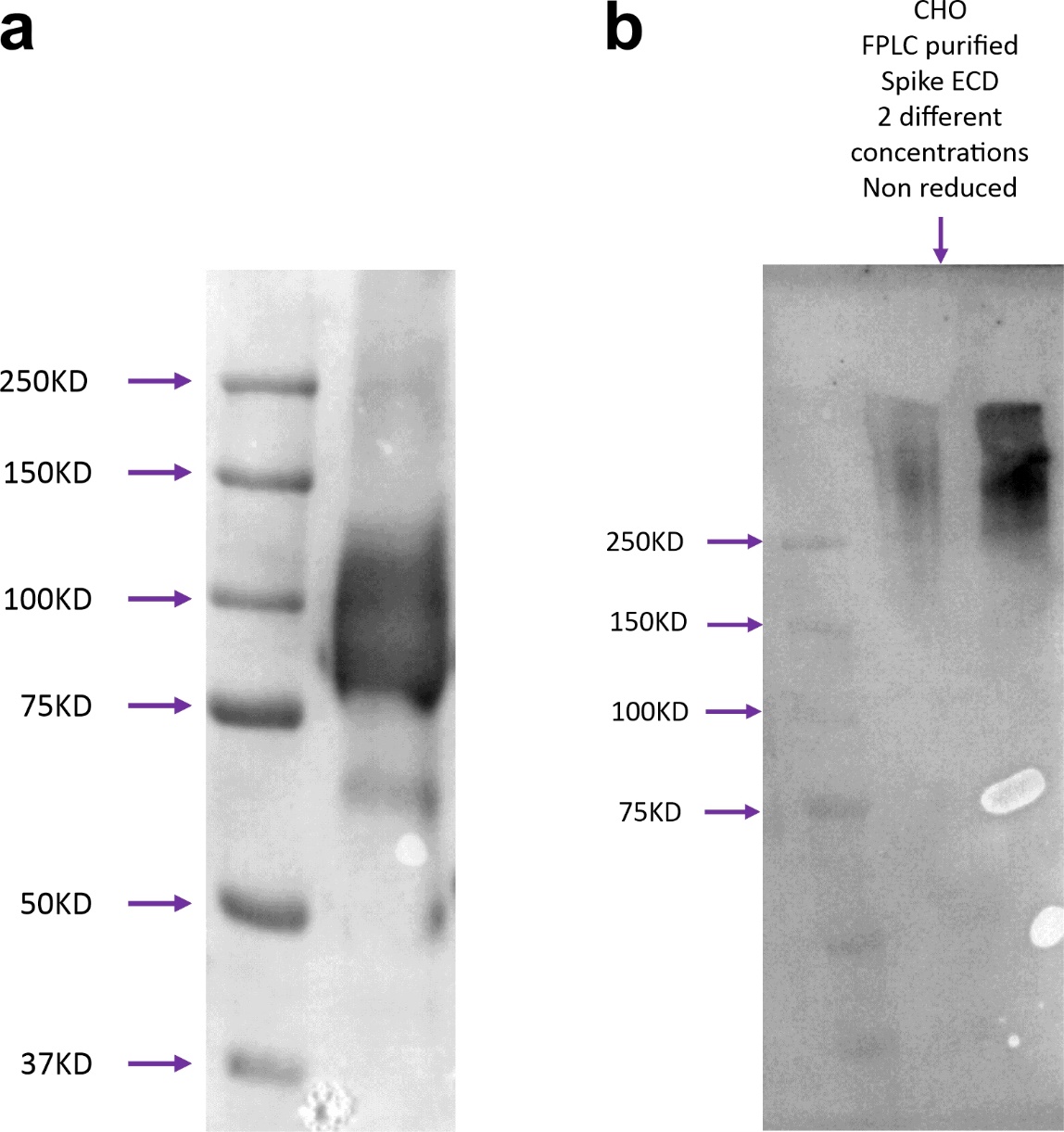


**Supplementary information, Figure S12**: Coomassie-stained SDS-page of purified **(a)** SARS-CoV-2-RBD and **(b)** SARS-CoV-2-ECD


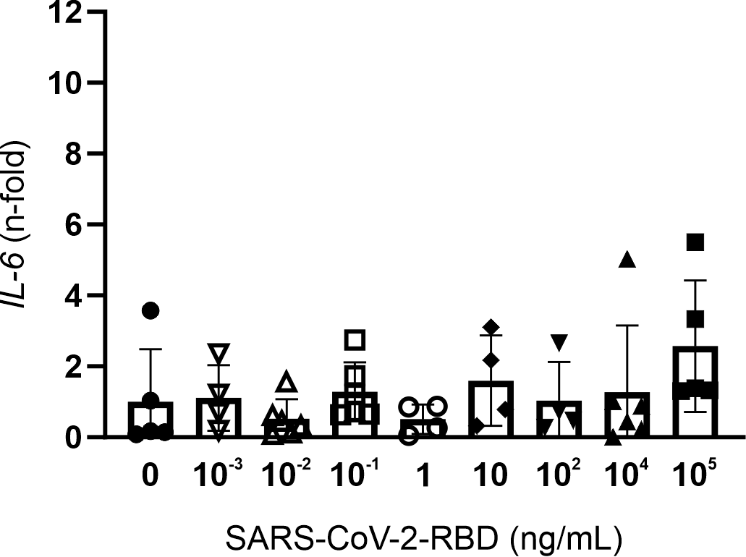


**Supplementary information, Figure S13**: Human THP-1 MΦ were treated with increasing concentrations of SARS-CoV-2-RBD. After 24h, IL-6 expression was quantified by qRT-PCR; n=4.


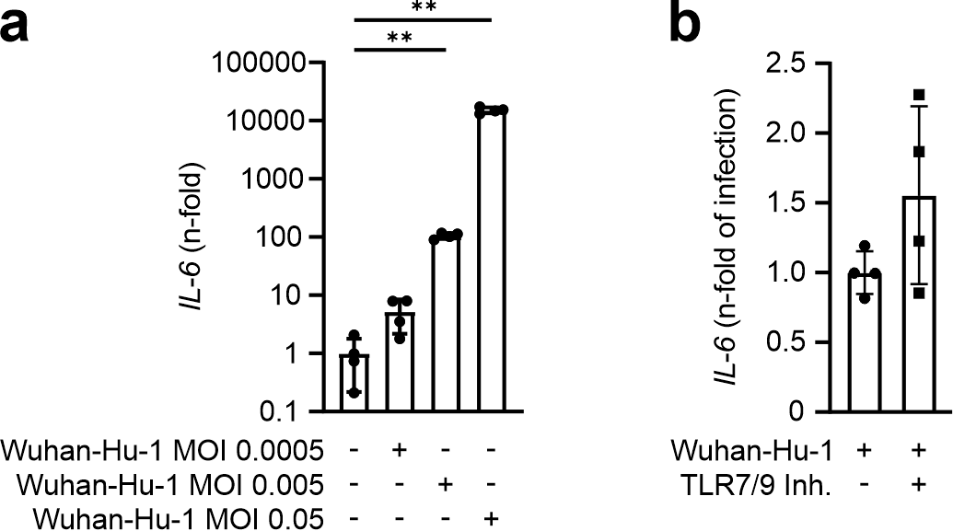


**Supplementary information, Figure S14:** **(a)** Human THP-1 MΦ were infected with increasing concentrations of Wuhan-Hu-1 strain. After 24h IL-6 expression was quantified by qRT-PCR.**;** **(b)** Human THP-1 MΦ were infected with Wuhan-Hu-1 strain and treated with an inhibitor of TLR7/9**.** After 24h IL-6 expression was quantified by qRT-PCR. TLR7/9 Inh. = HCQ sulfate; **P < 0.005; n=4.


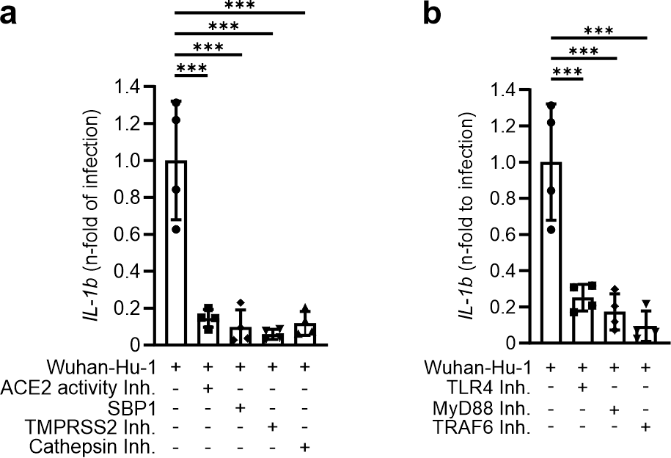


**Supplementary information, Figure S15:** Human THP-1 MΦ were infected with Wuhan-Hu-1 strain (MOI 0.005) and treated with inhibitors of virus entry **(a)** and of theTLR4 pathway **(b).** After 24h IL-1b expression was quantified by qRT-PCR. ACE2 activity inh. = MLN-4760, TMPRSS2 inh = Camostat mesylate, Cathepsin inh. = E64d, SBP1 = Spike binding protein, human ACE2 derived 23-amino acid peptide for competitive binding of S protein, TLR4 Inh. = TAK-242, MyD88 Inh. = NBP2-29328, TRAF6 Inh. = C25-140; ***P<0.001; n=4.


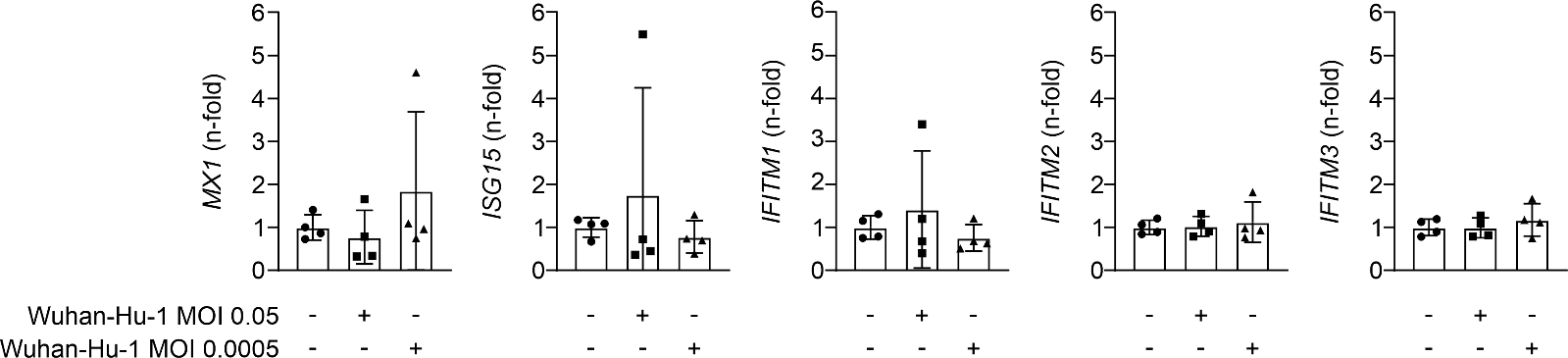


**Supplementary information, Figure S16:** Human THP-1 MΦ were infected with increasing concentrations of the Wuhan-Hu-1 strain. After 24h, expression of MX1, ISG15, IFITM1, IFITM2 and IFITM3 was quantified by qRT-PCR; n=4.

**
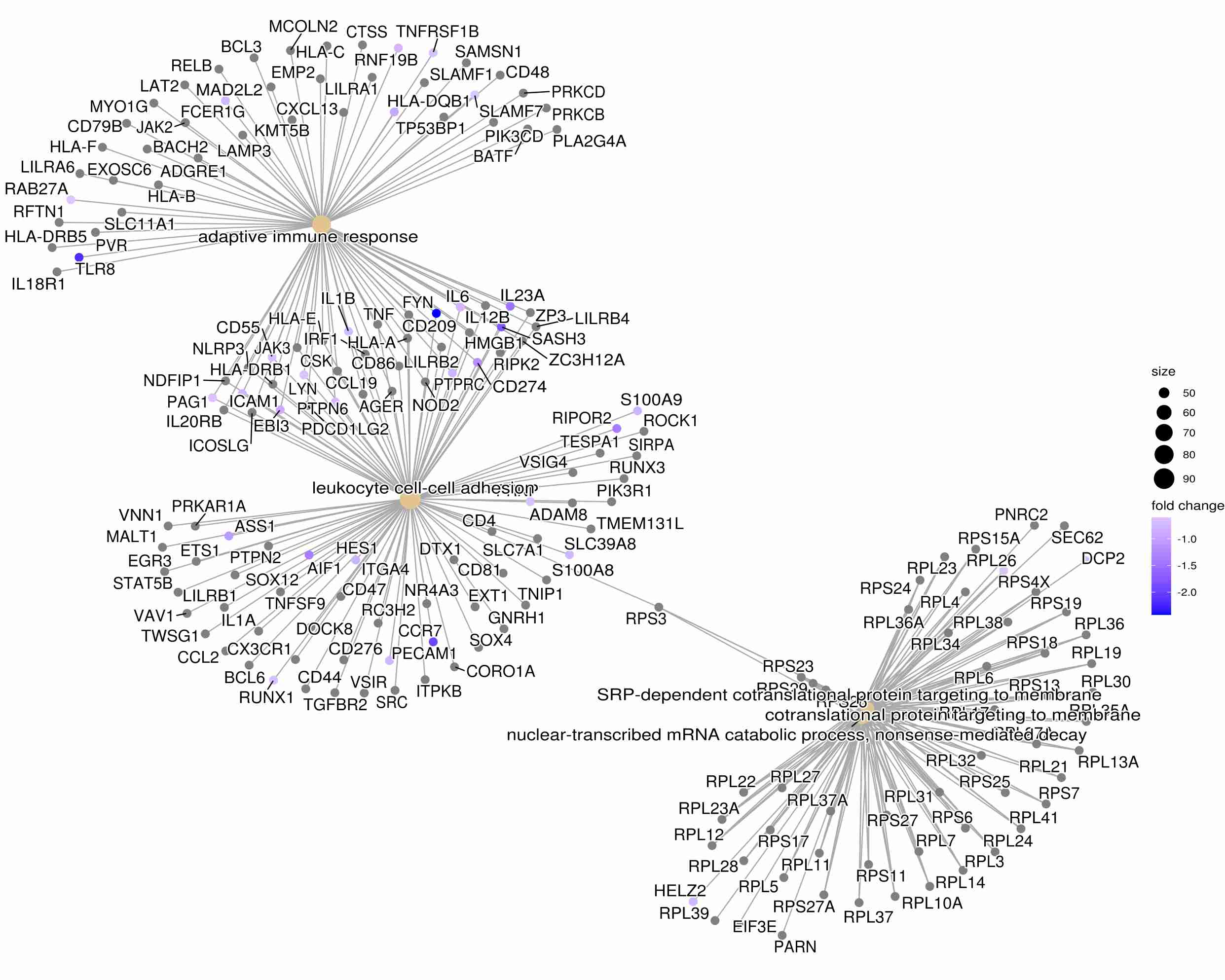
**

**Supplementary information, Figure S17:** Pathway enrichment analysis of human THP-1 MΦ infected with viable SARS-CoV-2 (Wuhan-Hu-1, MOI 0.005) with and without TLR4 inhibitor TAK-242 (10µM). Colored dots indicate genes downregulated by TAK-242.
